# Severe Cognitive Decline in Long-term Care Is Related to Gut Microbiome Production of Metabolites Involved in Neurotransmission, Immunomodulation, and Autophagy

**DOI:** 10.1093/gerona/glaf053

**Published:** 2025-03-28

**Authors:** Andrew P Shoubridge, Lucy Carpenter, Erin Flynn, Lito E Papanicolas, Josephine Collins, David Gordon, David J Lynn, Craig Whitehead, Lex E X Leong, Monica Cations, David P De Souza, Vinod K Narayana, Jocelyn M Choo, Steve L Wesselingh, Maria Crotty, Maria C Inacio, Kerry Ivey, Steven L Taylor, Geraint B Rogers

**Affiliations:** Microbiome and Host Health Program, South Australian Health and Medical Research Institute, Adelaide, South Australia, Australia; Infection and Immunity, Flinders Health and Medical Research Institute, College of Medicine and Public Health, Flinders University, Bedford Park, South Australia, Australia; Microbiome and Host Health Program, South Australian Health and Medical Research Institute, Adelaide, South Australia, Australia; Infection and Immunity, Flinders Health and Medical Research Institute, College of Medicine and Public Health, Flinders University, Bedford Park, South Australia, Australia; Microbiome and Host Health Program, South Australian Health and Medical Research Institute, Adelaide, South Australia, Australia; Microbiome and Host Health Program, South Australian Health and Medical Research Institute, Adelaide, South Australia, Australia; Infection and Immunity, Flinders Health and Medical Research Institute, College of Medicine and Public Health, Flinders University, Bedford Park, South Australia, Australia; SA Pathology, Adelaide, South Australia, Australia; Microbiome and Host Health Program, South Australian Health and Medical Research Institute, Adelaide, South Australia, Australia; SA Pathology, Adelaide, South Australia, Australia; Department of Microbiology and Infectious Diseases, Flinders Medical Centre, Bedford Park, South Australia, Australia; Infection and Immunity, Flinders Health and Medical Research Institute, College of Medicine and Public Health, Flinders University, Bedford Park, South Australia, Australia; Computational & Systems Biology Program, South Australian Health and Medical Research Institute, Adelaide, South Australia, Australia; Department of Rehabilitation, Aged and Palliative Care, Flinders Medical Centre, Flinders University, Bedford Park, South Australia, Australia; Registry of Senior Australians, South Australian Health and Medical Research Institute, Adelaide, South Australia, Australia; SA Pathology, Adelaide, South Australia, Australia; Registry of Senior Australians, South Australian Health and Medical Research Institute, Adelaide, South Australia, Australia; College of Education, Psychology and Social Work, Flinders University, Bedford Park, South Australia, Australia; Bio21 Institute and Department of Biochemistry and Molecular Biology, University of Melbourne, Parkville, Victoria, Australia; Bio21 Institute and Department of Biochemistry and Molecular Biology, University of Melbourne, Parkville, Victoria, Australia; Microbiome and Host Health Program, South Australian Health and Medical Research Institute, Adelaide, South Australia, Australia; Infection and Immunity, Flinders Health and Medical Research Institute, College of Medicine and Public Health, Flinders University, Bedford Park, South Australia, Australia; Microbiome and Host Health Program, South Australian Health and Medical Research Institute, Adelaide, South Australia, Australia; Infection and Immunity, Flinders Health and Medical Research Institute, College of Medicine and Public Health, Flinders University, Bedford Park, South Australia, Australia; Registry of Senior Australians, South Australian Health and Medical Research Institute, Adelaide, South Australia, Australia; Department of Rehabilitation, Aged and Palliative Care, Flinders Medical Centre, Flinders University, Bedford Park, South Australia, Australia; Registry of Senior Australians, South Australian Health and Medical Research Institute, Adelaide, South Australia, Australia; Registry of Senior Australians, South Australian Health and Medical Research Institute, Adelaide, South Australia, Australia; Allied Health and Human Performance, University of South Australia, Adelaide, South Australia, Australia; Microbiome and Host Health Program, South Australian Health and Medical Research Institute, Adelaide, South Australia, Australia; Department of Nutrition, Harvard University T.H. Chan School of Public Health, Boston, Massachusetts, USA; Microbiome and Host Health Program, South Australian Health and Medical Research Institute, Adelaide, South Australia, Australia; Infection and Immunity, Flinders Health and Medical Research Institute, College of Medicine and Public Health, Flinders University, Bedford Park, South Australia, Australia; Microbiome and Host Health Program, South Australian Health and Medical Research Institute, Adelaide, South Australia, Australia; Infection and Immunity, Flinders Health and Medical Research Institute, College of Medicine and Public Health, Flinders University, Bedford Park, South Australia, Australia; (Biological Sciences Section)

**Keywords:** Aged care, Cognitive impairment, Microbiome, Microbiome–gut–brain axis

## Abstract

Aging-associated cognitive decline affects more than half of those in long-term residential aged care. Emerging evidence suggests that gut microbiome–host interactions influence the effects of modifiable risk factors. We investigated the relationship between gut microbiome characteristics and severity of cognitive impairment (CI) in 159 residents of long-term aged care. Severe CI was associated with a significantly increased abundance of proinflammatory bacterial species, including *Methanobrevibacter smithii* and *Alistipes finegoldii*, and decreased relative abundance of beneficial bacterial clades. Severe CI was associated with increased microbial capacity for methanogenesis, and reduced capacity for synthesis of short-chain fatty acids, neurotransmitters glutamate and gamma-aminobutyric acid, and amino acids required for neuroprotective lysosomal activity. These relationships were independent of age, sex, antibiotic exposure, and diet. Our findings implicate multiple gut microbiome–brain pathways in aging-associated cognitive decline, including inflammation, neurotransmission, and autophagy, and highlight the potential to predict and prevent cognitive decline through microbiome-targeted strategies.

Progressive loss of cognitive function is a common feature of aging and is not limited to those with dementia (1–4). Contributory pathologies, often occurring in combination, include ischemic or hemorrhagic infarcts within the brain (characteristic of vascular dementia) (5,6), the accumulation of amyloid plaques and neurofibrillary tangles (characteristic of Alzheimer’s disease) (7,8), and the development of abnormal collections of alpha-synuclein protein within diseased brain neurons (characteristic of Lewy body dementia) (9,10). While these pathophysiological processes are increasingly well characterized, the factors that contribute to them and their relationship to external risk exposures remain poorly understood.

In addition to genetic factors (11–16), modifiable risk factors associated with dementia have been identified. Modifiable risk factors include exposures (smoking, excessive alcohol consumption, physical inactivity, air pollution, diet), health conditions (hypertension, obesity, depression, diabetes, traumatic brain injury, hearing impairment), and social factors (less education and low social contact) (17). Together, these modifiable risk factors are estimated to account for 40% of dementia incidence (17). Identifying how such factors influence the pathophysiology of aging-associated cognitive impairment (CI) is essential to the development of effective prevention and treatment.

The gut microbiome influences neurophysiology, central nervous system, and cognitive function through discrete bidirectional pathways, collectively termed the microbiome–gut–brain axis (18–21). These pathways include the microbial synthesis of neurotransmitters, such as gamma-aminobutyric acid (GABA), noradrenaline, dopamine, and serotonin (22,23), the modulation of systemic immunity (24,25), and metabolism of essential amino acids, such as tyramine and tryptophan (26,27). They also involve production of immune and metabolically active metabolites, such as short-chain fatty acids (SCFAs) and 4-ethylphenylsulfate, and activation of nerve growth factor, glial-derived neurotrophic factor, and brain-derived neurotrophic factor secretion (28,29). Such microbial traits have the potential to contribute substantially to the development of neurological diseases, including Alzheimer’s (30,31), Huntington’s (32–34), and Parkinson’s diseases (35,36).

Aging-associated gut microbiome characteristics (37,38) are linked to progressive frailty and cognitive decline (4,38–40). External exposures that disrupt the microbiome, such as antibiotics, can further contribute to altered neurological homeostasis and poorer cognitive outcomes (41–43). In contrast, dietary interventions that alter the composition of the gut microbiome in a beneficial manner can result in improvements in cognitive function (44). Such findings suggest that the relationship between the gut microbiome and host neurophysiology may provide a basis to predict and/or prevent the onset and progression of aging-associated cognitive decline. Potential causality in these relationships is suggested by studies that have successfully recapitulated impairment of memory and synaptic plasticity following fecal microbiota transplant from aged mice to younger mice (45).

Our aim was to explore whether the severity of CI experienced by residents of long-term aged care facilities (sometimes referred to as nursing homes, care homes, or residential aged care facilities) is associated with characteristics of the gut microbiome, and if so, whether such relationships might provide mechanistic insight into CI pathogenesis.

## Method

### Study Design, Cohort, and Data Collection

The Generating evidence on Resistant bacteria in the Aged Care Environment (GRACE) study (www.gracestudy.com.au) was a cohort study supported by the Australian Medical Research Future Fund (Grant No. GNT1152268). Ethical approval for the study was obtained from the Southern Adelaide Clinical Human Research Ethics Committee (HREC/18/SAC/244). The GRACE study investigated the carriage and transfer of resistant bacteria in long-term aged care facilities and was conducted between 2018 and 2020. GRACE enrolled 279 residents in 5 long-term aged care facilities in metropolitan South Australia. Anonymized participant data, including assessments of cognition and behavior, were collected via an entry into care funding assessment (Aged Care Funding Instrument [ACFI]), in addition to medications prescribed via the Pharmaceutical Benefits Scheme (PBS) (46).

### Assessment of CI

The *Cognitive Skills* component of the ACFI was used as a basis for assessment of CI. This cognitive skills component assesses a person’s cognitive abilities in everyday activities, including memory, self-care, and orientation (47,48), as defined via the Psychogeriatric Assessment Scales – Cognitive Impairment Scales (PAS-CIS) method (49). Where individuals were unable to undertake the PAS-CIS test, for example, non-English speaking, sensory impairment, or severe CI beyond the scope of the instrument, the ACFI cognitive skills assessment was based on a clinical report by a registered health professional (47). The ACFI cognitive skills component utilized the PAS-CIS and/or clinical reports to rate an individual’s level of CI as none or minimal (PAS-CIS = 0–3), mild (4–9), moderate (10–15), or severe (16–21).

### CI Cohort

GRACE participants were categorized according to their cognitive skills rating, as defined in the ACFI. Participants were excluded if: (1) their cognitive skills assessment was not completed or missing, (2) the date of stool collection was not known, (3) the date of the cognitive assessment was not known, (4) the participant was diagnosed with a developmental or intellectual disability, or (5) the period between cognitive skills assessment and stool sample collection was not known or was deemed an outlier (>1 462 days as determined using the Robust regression and Outlier [ROUT] removal method (50)). A total of 45 participants were excluded (detailed in Supplementary Figure 1). Participants with dementia and a missing PAS-CIS rating were imputed the median PAS-CIS value from their cognitive skills assessment group. Mental and behavioral diagnoses of dementia, depression, and delirium were ascertained from the ACFI, where a documented diagnosis from a medical practitioner was provided.

### Fecal Collection, DNA Extraction, Metagenomic Sequencing, and Bioinformatics

Stool sample was collected and stored using Norgen Stool Nucleic Acid Collection and Preservation Tubes (Norgen Biotek, Thorold, ON, Canada), and microbial DNA was extracted using the PowerLyzer PowerSoil DNA Isolation Kit (Qiagen, Hilden, Germany) as described previously (46). Indexed, paired-end DNA libraries were prepared using the Nextera XT DNA Library Prep Kit (Illumina, San Diego, CA), as per manufacturer’s instructions. Samples were sequenced at a depth of 5 Gb on an Illumina Novaseq platform with 150 bp paired-end reads. Forward and reverse sequences were quality-filtered using Trimmomatic (v0.39), and human reads were removed with Bowtie (v2.3.5.1) against the NCBI human reference genome release GRCh38 (51,52). Taxonomic relative abundance was assigned using MetaPhlAn (v3.0) (53), while microbial metabolic pathway abundance was assigned using HUMAnN (v3.0) against the MetaCyc database (53). Sequence data have been entered into the European Nucleotide Archive (ENA) at EMBL-EBI under accession number PRJEB51408.

### Microbiome Characterization

The taxonomic relative abundance at the species level was used to generate alpha diversity (within-group) and beta diversity (between-group) measures. Alpha diversity measures included Pielou’s evenness (*J*′: a score between 0 and 1 where scores are influenced more by the evenness of abundant species), the Shannon–Wiener diversity (*H*′): a score of the number and equal representation of different types of species (54), and species richness (*d*: total number of unique species identified per participant) and were generated using the “vegan” R package (55).

The Bray–Curtis index was calculated to compare microbiome similarity between groups (beta diversity), using square-root transformed species relative abundance data (PRIMER 6 [v6.1.16]). For sensitivity analysis, weighted and unweighted UniFrac distance matrices (56) were calculated using the “calculate_unifrac” MetaPhlAn R script (53). Nonmetric multidimensional scaling (nMDS) plots for all beta diversity measures were generated using the “vegan” package in R and visualized using “ggplot2.”

### Microbial Functional Profiling

The functional capacity of the gut microbiota was defined by the genetically encoded functional traits detected within the metagenome. These MetaCyc pathways from HUMAnN were filtered to only analyze those present in >30% of participants. Two functional profiling analyses were performed: an untargeted analysis of all filtered pathways (*n* = 400) and a targeted analysis based on pathways with a hypothesized functional role in CI (*n* = 70). These included pathways involved in neurotransmitter biosynthesis (*n* = 2), SCFA production (*n* = 25), and amino acid biosynthesis (*n* = 43).

### Metabolite Profiling

As a confirmatory analysis of microbial functional capacity, the metabolomic profile of a randomly selected subgroup of individuals (*n* = 35; *n* = 11–12/group) was established. Stool metabolite analysis was performed on an Agilent 1200 series high-performance liquid chromatography system (Agilent Technologies) (methods modified from (57,58) and detailed in Supplementary Methods). Briefly, metabolite extraction and analysis were performed separately for SCFAs and polar metabolites. SCFA analysis was performed using an Agilent 6490 series triple quadrupole mass spectrometer (Agilent Technologies), while polar metabolites (a screen for 165 low-molecular-weight metabolites, eg, amino acids) were analyzed using an Agilent 6545 series quadrupole time-of-flight mass spectrometer (Agilent Technologies). Resultant data matrices were imported to the web-based platform MetaboAnalyst (v5.0) for quality control checks. SCFA data were normalized to internal standards, and polar metabolite data were log-transformed and median-normalized.

### Covariates

Covariates were: days since cognitive assessment (below or equal/above the median), age (low, medium, or high tertile), sex (male or female), medication history (PBS data available or unavailable), medications that affect gastrointestinal health and are prevalent in aged care facilities (antibiotic use [yes or no]; proton pump inhibitor use [yes or no]; opioid use [yes or no]; laxative use [yes or no]), meal texture at time of cognitive assessment (regular or soft/smooth), and liquid texture at time of cognitive assessment (normal/thin or thick). Medication use was defined as 2 or more supplies to a resident within 90 days prior to stool collection.

### Statistical Analysis

Both unadjusted and multivariate regression models were applied in all analyses. Multivariate-adjusted models accounted for time between cognitive assessment and stool collection, age, sex, antibiotics, proton pump inhibitors, opioids, laxatives, meal texture, and liquid texture (as detailed above).

Beta diversity analysis was performed using permutational multivariate analyses of variance (PERMANOVA) on Bray–Curtis, weighted, and unweighted UniFrac distance matrices in an unrestricted permutation of raw data. Only the Bray–Curtis metric was assessed with the multivariate-adjusted model. PERMANOVA analyses were performed using PRIMER 6, with 9999 permutations.

Within-individual microbiome variables included alpha diversity, phyla-level relative abundance (only those detected in >30% of participants), species-level relative abundance (only those detected in >30% of participants), metabolic pathway abundance (only those detected in >30% of participants), and metabolite intensities. All within-individual variables were converted to groups consisting of: zero values, tertile 1, tertile 2, and tertile 3. Ordinal logistic regression was performed to assess the effect of CI on microbiome variables using the “MASS” function in R. The odds ratios (ORs) and 95% confidence intervals for the coefficients of the regression models were calculated and tested for statistical significance (*p* < .05) as CI severity increased, using the PAS-CIS score of CI as both a continuous variable (score 0–21) and as a categorical variable (classification of mild, moderate, and severe) for the predictor variable. False discovery rate (FDR) multiple hypothesis testing was conducted with the Benjamini and Hochberg method across all profiles using the “p.adjust” function in R, at a significant threshold of 0.05. Correlations between microbial functional capacity and detected metabolites were calculated by 2-tailed Spearman correlations and tested for statistical significance (*p* < .05).

## Results

The study group of 159 participants did not differ from the original GRACE cohort in any of the assessed characteristics (Supplementary Table 1). CI was classified as mild in 46 individuals (28.9%) with a median PAS-CIS score of 6.6 (interquartile range [IQR] = 5.0–8.0), moderate in 58 (36.5%; PAS-CIS median = 11.0; IQR = 11.0–12.8), and severe in 55 (34.6%; PAS-CIS median = 18.0; IQR = 17.0–18.8) (Table 1; Supplementary Figure 2A). Participant age, sex ratio, time since cognitive assessment, and use of antibiotics, opioids, and laxatives did not differ significantly between CI severity categories (*p* > .05; Supplementary Figure 2B–F). However, the number of days that an individual had been residing in long-term aged care was significantly higher for those in the severe CI group (median = 939 days; IQR = 219–854) compared to the mild CI group (median = 500 days; IQR = 130–627; *p* < .05). Proton pump inhibitor usage was significantly lower in the severe CI group (*p* < .05).

**Table 1.** Study Cohort Characteristics by Severity of Cognitive Impairment

Demographic	Mild (*n* = 46)	Moderate(*n* = 58)	Severe(*n* = 55)	Total (*n* = 159)
Age (years): median (IQR)	87.5 (81.3, 93.6)	90.3 (83.7, 95.0)	87.9 (82.0, 93.0)	88.7 (82.1, 93.5)
Sex: *n* (%)				
Female	27 (58.7)	42 (72.4)	40 (72.7)	109 (68.6)
Male	19 (41.3)	16 (27.6)	15 (27.3)	50 (31.4)
PAS-CIS: median (IQR)	6.6 (5.0, 8.0)	11.0 (11.0, 12.8)	18.0 (17.0, 18.8)	10.8 (7.0, 18.0)
Time since entry to facility (days): median (IQR)	500 (253.0, 947.5)	704 (299.0, 983.0)	962 (502.0, 1198.0)	681 (360.0, 1015.0)
Dementia diagnosis: % (*n*)*	8.7 (4)	56.9 (33)	96.4 (53)	56.6 (90)
Memory support unit^†^: *n* (%)	0 (0.0)	4 (6.9)	19 (34.5)	23 (14.5)
Meal texture: *n* (%)				
Regular	38 (82.6)	44 (75.9)	30 (54.5)	112 (70.4)
Soft/smooth	8 (17.4)	14 (24.1)	25 (45.5)	47 (29.6)
Liquid texture: *n* (%)				
Normal/thin	42 (91.3)	54 (93.1)	49 (89.1)	145 (91.2)
Thick	4 (8.7)	4 (6.9)	6 (10.9)	14 (8.8)
Antibiotics (at least 2 supplied^‡^): *n* (%)	10 (21.7)	7 (12.1)	5 (9.1)	22 (13.8)
Proton pump inhibitors (at least 2 supplied^‡^): *n* (%)	23 (50.0)	23 (39.7)	12 (21.8)	58 (36.5)
Opioids (at least 2 supplied^‡^): *n* (%)	8 (17.4)	12 (20.7)	14 (25.5)	34 (21.4)
Laxatives (at least 2 supplied^‡^): *n* (%)	6 (13.0)	6 (10.3)	9 (16.4)	21 (13.2)

*Notes*: IQR = interquartitle range; PAS-CIS = Psychogeriatric Assessment Scales – Cognitive Impairment Scales.

*Extracted from Aged Care Funding Instrument data.

^†^Memory support units (also known as dementia units/wards, memory care, or special care units) are secure areas within long-term care facilities specially designed to accommodate residents with dementia.

^‡^Medication use defined as 2 or more supplies of the same medication within 90 days prior to stool collection.

Of those with severe CI, 53/55 (96.4%) had a concurrent diagnosis of dementia, while 33/58 (56.9%) with moderate CI had a dementia diagnosis, and 4/46 (8.7%) for those with mild CI.

### Gut Microbiome Characteristics Differ by CI Severity

**Figure 1.**
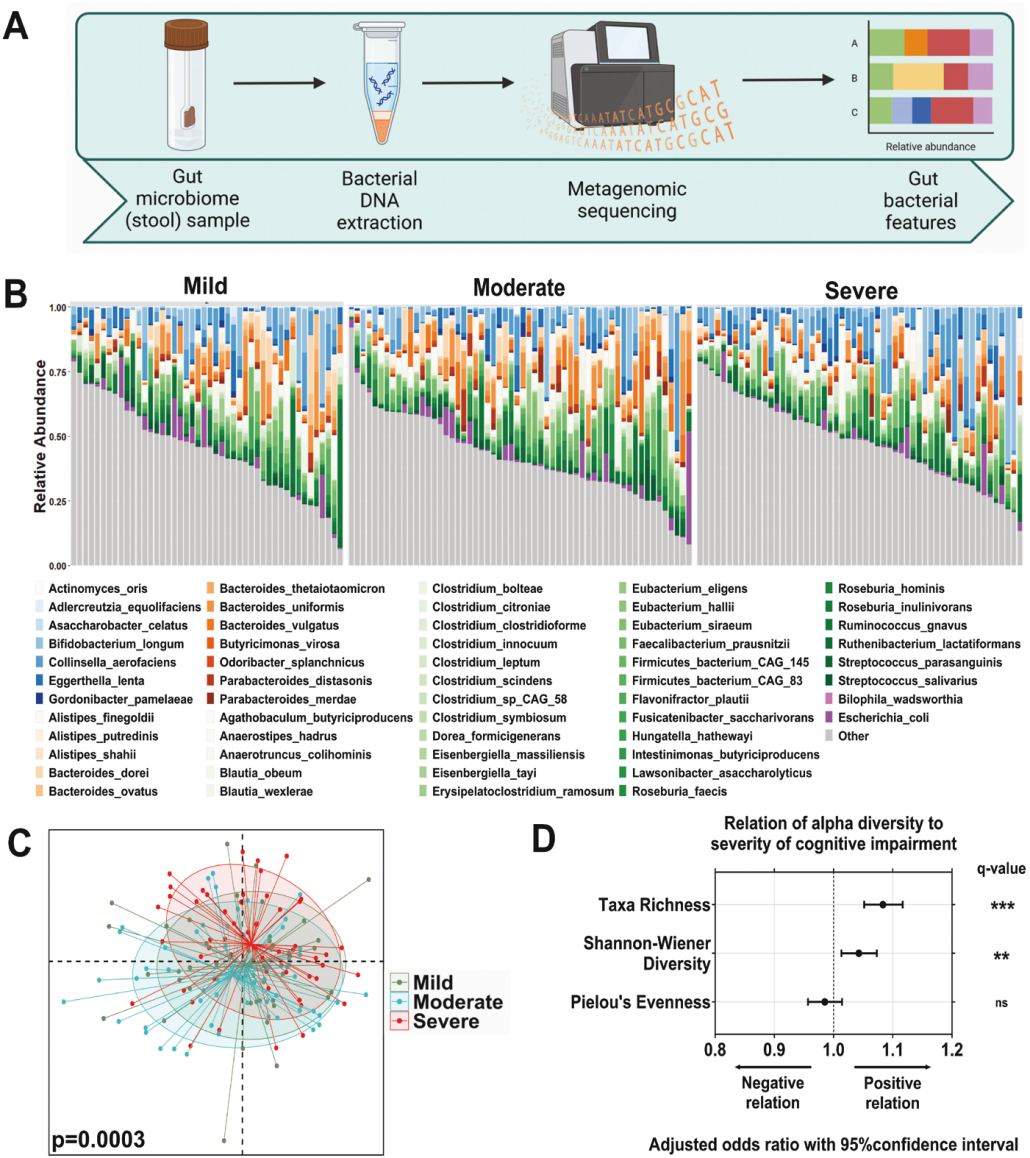
Gut microbiome of residents of long-term aged care facilities stratified by cognitive impairment (CI). A) Characterization of the gut microbiome of long-term aged care residents with cognitive impairment determined by metagenomic sequencing of collected stool samples. B) Taxa bar plot of core species grouped by cognitive impairment severity (present in >60% of participants). Species colored by phyla: Actinomycetota = blues; Bacteroidota = oranges; Bacillota = greens; Pseudomonadota = purples; non-core species (other) = gray. C) Nonmetric multidimensional scaling plot of Bray–Curtis similarity matrix, grouped by CI severity (mild, *n* = 46; moderate, *n* = 58; severe, *n* = 55), showing significant divergence between CI groups following multivariate analysis (*p*(perm) = .0003). D) Odds ratio and 95% confidence interval of effect of CI severity on microbiome diversity (taxa richness, Shannon–Wiener diversity, and Pielou’s evenness), following multivariate analysis. Multivariate analysis treated CI severity as a continuous variable and adjusted for time since CI assessment, age, sex, antibiotic use, proton pump inhibitor use, opioid use, laxative use, recorded medical history, meal texture, and liquid texture. ns = not significant; **q < 0.01; ***q < 0.001 for adjusted *p*-values following FDR correction.

Gut microbiome characterization of long-term aged care residents with CI was determined by metagenomic sequencing of collected stool samples (Figure 1A). A total of 11 bacterial phyla were detected across the 159 stool samples, consisting of 186 genera (586 species). The composition and distribution of taxa was broadly similar with previous studies of aged populations (37,59), with high representation of *Eggerthella lenta*, *Escherichia coli*, *Faecalibacterium prausnitzii*, and *Clostridium* species (Figure 1B), and genera within the Bacteroidota (formerly Bacteroides) and Bacillota (Firmicutes) phyla (Supplementary Figure 3).

Following adjustment for time since cognitive assessment, age, sex, medication use, and diet, the fecal microbiota composition differed significantly between mild, moderate, and severe CI (*p*(perm) = .0003; *R*^2^ = 2.21%; Figure 1C; Table 2). This difference was greatest between severe CI and mild CI (*p*(perm) = .0023), and severe CI and moderate CI (*p*(perm) = .0003), and consistent with the unadjusted model (Table 2). Repeated analysis using weighted and unweighted UniFrac dissimilarity did not identify significant intergroup differences, apart from between mild and moderate CI groups using weighted UniFrac dissimilarity (unadjusted *p*(perm) = .037; Supplementary Figure 4).

**Table 2.** Permutational ANOVA of the Gut Microbiome by Severity of Cognitive Impairment

	Unadjusted Model			Multivariate-adjusted Model*		
Main Test	Pseudo-*F* Ratio	*R* ^2^	*p*(perm)^†^	Pseudo-*F* Ratio	*R* ^2^	*p*(perm)^†^
Cognitive impairment^‡^	1.9646	0.0246	**.0002**	1.815	0.0221	**.0003**

Pairwise Test	*t*		*p*(perm)^†^	*t*		*p*(perm)^†^
Mild vs moderate	1.0596		.2583	1.1135		.1478
Mild vs severe	1.4474		**.0017**	1.4228		**.0023**
Moderate vs severe	1.6304		**.0002**	1.5265		**.0003**

*Notes*:

Bold values indicate statistical significance.*Time since cognitive impairment assessment + age + sex + antibiotic use + proton pump inhibitor use + opioid use + laxative use + recorded medication history + meal texture + liquid texture.

^†^Permutation *p*-value generated with a PERMANOVA.

^‡^Degrees of freedom = 2.

Analysis of microbiota diversity identified a positive association between CI severity and taxa richness (OR: 1.08 [95% confidence interval 1.05, 1.12], *q* < 0.001) and Shannon–Wiener diversity (OR: 1.043 [1.013, 1.073], *q* < 0.05; Figure 1D). However, there was no association between CI severity and Pielou’s evenness (OR: 0.985 [0.957, 1.014], *q* > 0.05; Figure 1D).

**Figure 2.**
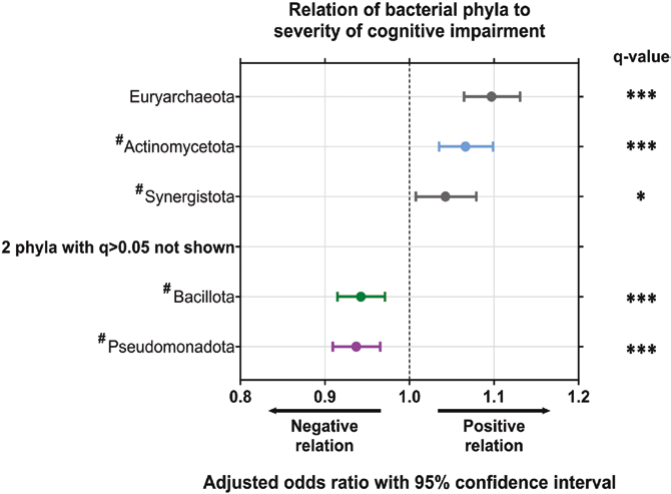
Phyla-level differences in the gut microbiome of residents of long-term aged care by cognitive impairment. Odds ratio and 95% confidence interval of effect of cognitive impairment severity on phyla relative abundance. Colors indicate bacterial phyla: blue = Actinomycetota; green = Bacillota; purple = Pseudomonadota; gray = non-core species. Performed by multivariate analysis treating CI severity as a continuous variable and adjusting for time since cognitive impairment assessment, age, sex, antibiotic use, proton pump inhibitor use, opioid use, laxative use, recorded medical history, meal texture, and liquid texture. ^#^Denotes phyla with recently amended names: Actinomycetota (Actinobacteria), Synergistota (Synergistetes), Bacillota (Firmicutes), and Pseudomonadota (Proteobacteria). *q < 0.05; ***q < 0.001 for adjusted *p*-values following FDR correction.

To assess whether specific taxa differed with severity and classification of CI, phylum-level and species-level relative abundances were assessed. Of the 7 phyla present in at least 30% of participants, 5 differed significantly with CI (Figure 2). Pseudomonadota (Proteobacteria) (OR: 0.937 [0.909, 0.965], *q* < 0.001) and Bacillota (OR: 0.943 [0.915, 0.971], *q* < 0.001) were lower with increasing CI severity (Figure 2). In contrast, Euryarchaeota (OR: 1.097 [1.065, 1.131], *q* < 0.001), Actinomycetota (Actinobacteria) (OR: 1.066 [1.035, 1.099], *q* < 0.001), and Synergistota (Synergistetes) (OR: 1.043 [1.008, 1.079], *q* < 0.05) were higher with increasing CI severity (Figure 2).

Bacterial species that were detected in at least 60% of participants, and with a relative abundance of at least 0.1%, were denoted as “core” taxa (Supplementary Table 2). Of the 586 microbial species identified across the entire cohort, 30 were identified as core in mild CI (Supplementary Figure 5A), 31 in moderate CI (Supplementary Figure 5B), and 29 in severe CI (Supplementary Figure 5C).

**Figure 3.**
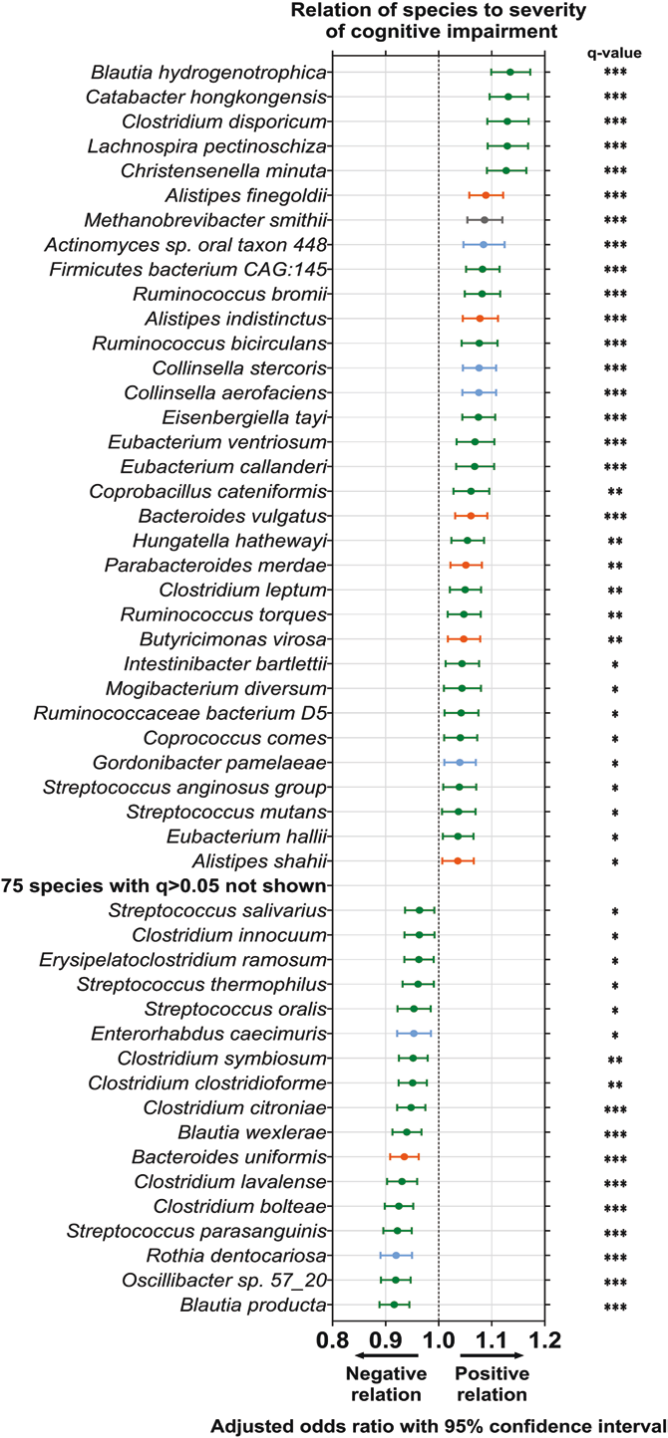
Species-level differences in the gut microbiome of residents of long-term aged care by cognitive impairment. Odds ratio and 95% confidence interval of effect of cognitive impairment severity on species relative abundance. Colors indicate bacterial phyla: blue = Actinomycetota; orange = Bacteroidota; green = Bacillota = green; gray = other. Performed by multivariate analysis treating CI severity as a continuous variable and adjusting for time since cognitive impairment assessment, age, sex, antibiotic use, proton pump inhibitor use, opioid use, laxative use, recorded medical history, meal texture, and liquid texture. ns = not significant; *q < 0.05; **q < 0.01; ***q < 0.001 for adjusted *p*-values following FDR correction. Mild, *n* = 46; moderate, *n* = 58; severe, *n* = 55.

Comparison of species relative abundances identified 50 species that differed significantly with CI severity scores (Figure 3). Notably, *Blautia hydrogentrophica* (OR: 1.135 [1.099, 1.173]), *Catabacter hongkongensis* (OR: 1.131 [1.096, 1.168]), and *Alistipes finegoldii* (OR: 1.089 [1.058, 1.121]) had the strongest positive association with CI severity (all *q* < 0.001, Figure 3). Further, *Collinsella aerofaciens* and *Methanobrevibacter smithii* were not only positively associated with CI severity (*q* < 0.001, Figure 3), they were also core species in residents categorized with severe CI, but not mild or moderate (Supplementary Table 2, Supplementary Figures 5 and 6). In contrast, *Bacteroides uniformis* (OR: 0.935 [0.908, 0.962]), *Blautia producta* (OR: 0.916 [0.888, 0.945]), and *Blautia wexlerae* (OR: 0.940 [0.913, 0.968]) were among those with the strongest inverse association with CI severity scores (all *q* < 0.001, Figure 3). *Faecalibacterium prausnitzii*, a species previously associated with health outcomes in aging (59), was also found to trend lower in this cohort with increasing CI severity score, but this did not achieve statistical significance (OR: 0.986 [0.958, 1.014], *q* = 0.421, Figure 3; *p* > .05, Supplementary Figure 6).

### The Functional Capacity and Output of the Gut Microbiota Differs With CI Severity

**Figure 4.**
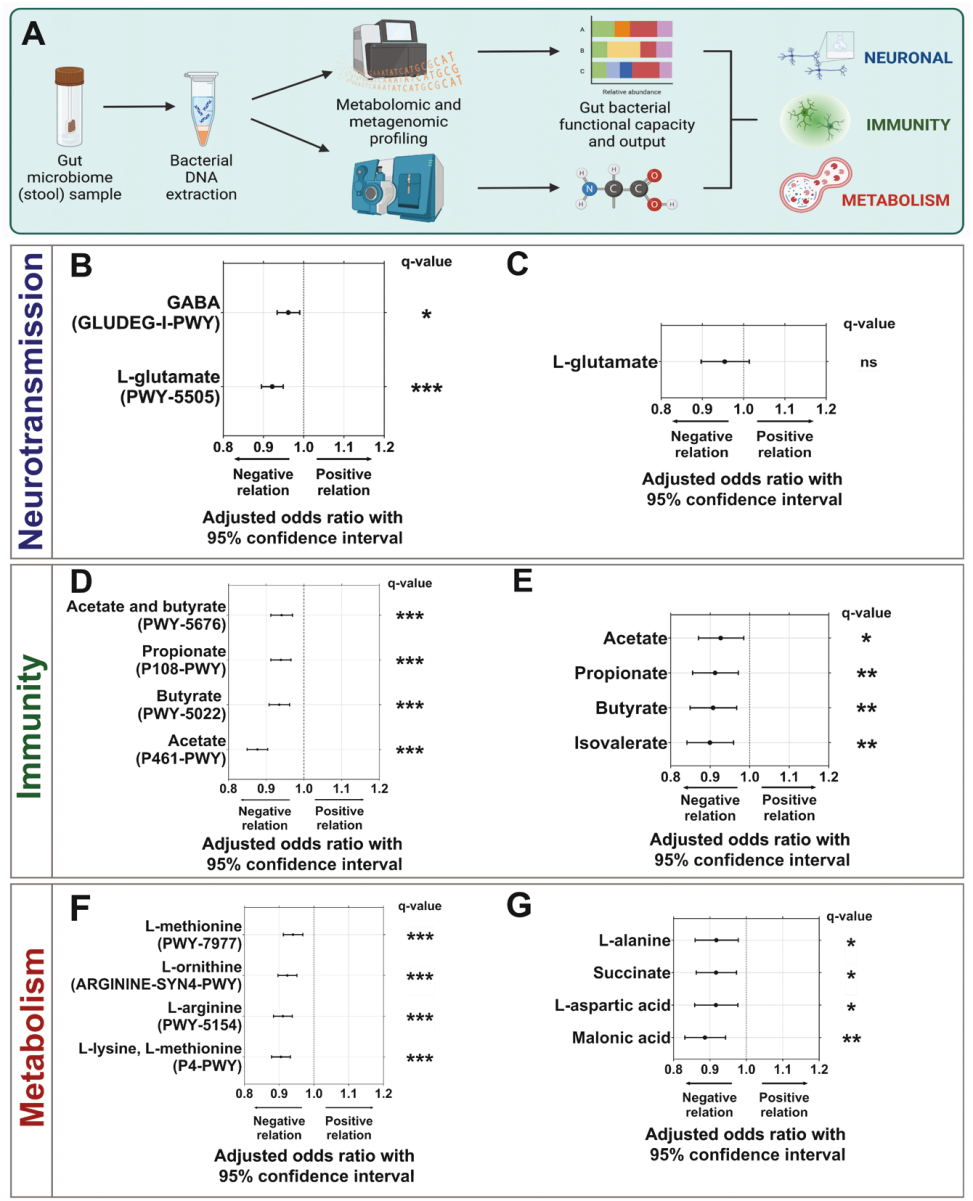
Specific functional differences relating to neurotransmission, immunity, and metabolism in the gut microbiome of residents of long-term aged care by cognitive impairment. A) Metagenomic and metabolomic profiling of microbiome functional capacity and output for long-term aged care residents with cognitive impairment in relation to neuronal communication (B, C), immunity (D, E), and metabolism (F–G). Odds ratio and 95% confidence interval of effect of cognitive impairment severity on functional pathway relative abundance and metabolite normalized abundance. Performed by multivariate analysis, treating CI severity as a continuous variable, and adjusting for time since cognitive impairment assessment, age, sex, antibiotic use, proton pump inhibitor use, opioid use, laxative use, recorded medical history, meal texture, and liquid texture. The abundance of key pathways and metabolites grouped by cognitive impairment severity involved in neurotransmission, immunomodulation, and metabolism are shown. ns = not significant; *q < 0.05; **q < 0.01; ***q < 0.001 for adjusted *p*-values following FDR correction. Pathways: mild, *n* = 46; moderate, *n* = 58; severe, *n* = 55. Metabolites: mild, *n* = 12; moderate, *n* = 11; severe, *n* = 12.

Differences in the functional capacity of the gut microbiota were identified with increasing CI severity. Four hundred MetaCyc pathways were detected in >30% of participants, of which, 70 were selected based on their potential influence on CI, including via mechanisms relating to neurotransmission, immunity, and metabolism. Metabolomic analysis of a subgroup of individuals (*n* = 35; *n* = 11–12/group) confirmed these findings (Figure 4A). A total of 165 polar metabolites were detected in stool samples from these participants, including 33 metabolites classed as amino acids, peptides, and analogues, 50 classed as lipids and lipid-like molecules, 18 classed as carbohydrates, and 64 classed within other categories (Supplementary Figure 7A), in addition to 9 SCFAs (Supplementary Figure 7B). Pathways inversely associated with CI severity included PWY-5505, a pathway essential to the production of the primary excitatory neurotransmitter glutamate (OR: 0.922 [0.895, 0.949], *q* < 0.001), and GLUDEG-I-PWY, a pathway essential to the production of the primary inhibitory neurotransmitter GABA (OR: 0.962 [0.934, 0.990], *q* < 0.05, Figure 4B, Supplementary Figure 8A). The metabolite and excitatory neurotransmitter glutamate was present at lower levels in individuals with more severe CI (Figure 4C). Similarly, pathways related to the production of immunomodulatory SCFAs, including acetate (P461-PWY; OR: 0.877 [0.850, 0.904], *q* < 0.001), propionate (P108-PWY; OR: 0.939 [0.913, 0.966], *q* < 0.001), and butyrate (PWY-5022; OR: 0.935 [0.908, 0.962], *q* < 0.001, Figure 4D) were also lower in relative abundance as CI severity increased and particularly in individuals classified with severe CI (Supplementary Figure 8B). The decrease in immune functional capacity corresponded with depleted levels of immunomodulating SCFA metabolites in individuals with severe CI, including for butyrate (*q* < 0.01), propionate (*q* < 0.01), and acetate (*q* < 0.05, Figure 4E; Supplementary Figures 8C and 9). Functional pathways related to the biosynthesis of amino acids that regulate key metabolic processes, such as autophagy, included l-arginine (PWY-5154; OR: 0.911 [0.884, 0.938], *q* < 0.001), l-lysine, l-threonine, and l-methionine (P4-PWY; OR: 0.905 [0.878, 0.932], *q* < 0.001), and were among the most depleted at higher CI severity (Figure 4F; Supplementary Figure 8D). Production of amino acid polar metabolites also decreased with increasing CI severity (Figure 4G; Supplementary Figures 8E and 9).

Further exploratory analysis across all functional pathways (*n* = 400) identified 271 statistically significantly altered pathways, with multiple pathways related to methanogenesis among those of greatest significance and higher relative abundance (*p* < .01, Supplementary Figure 8D; *q* < 0.001, Supplementary Table 3). The relative abundances of metagenomic pathways and the levels of associated metabolites were also positively correlated (Supplementary Figure 10).

## Discussion

We report significant associations between characteristics of the fecal microbiome and the severity of CI in residents of long-term aged care facilities. Microbiome CI severity-associated traits were identified even after adjustment for age, sex, prior medication exposure, and diet. Individuals with more severe CI exhibited a greater representation of the Actinomycetota phylum and *Methanobrevibacter smithii*, and a lower prevalence of *Bacteroides uniformis*, a reduced capacity for synthesis of SCFAs, neurotransmitters (glutamate and GABA), and amino acids that are essential for autophagy, and an increased capacity for methanogenesis. These findings identify microbial factors potentially influencing aging-associated cognitive decline and present opportunities for prediction and treatment of CI.

Changes in intestinal microbiology can influence neuroplastic changes in the brain via a range of mechanisms (18–21). Many of these pathways relate to the production of specific factors by the gut microbiota, including the biosynthesis of immunomodulatory metabolites and neurotransmitters (24,60), amino acid metabolism (61,62), and the release of proinflammatory cytokines (60,63,64).

We assessed the potential influence of the gut microbiome of participants to neurophysiology through 2 complementary strategies. The first was the analysis of the metagenome, representing the functional capacity of microbes within the gut to produce particular metabolites. The second was a confirmatory analysis of the fecal metabolome, representing the output of the combined metabolic activity of the gut microbiota. Each of these processes identified factors that were significantly associated with CI severity, and notably, positive correlations between metabolite levels and the prevalence of genes involved in their biosynthesis was widespread.

A lower capacity for microbial biosynthesis of the neurotransmitters, glutamate and GABA, was evident in those with more severe CI. Levels of both factors have been associated with CI previously (9,65–68). The gut microbiome mediates neurological homeostasis via multiple key pathways, including through metabolism and production of neurotransmitters, such as glutamate, GABA, dopamine, and serotonin. These neurotransmitters can then directly innervate intestinal neural pathways or circulate peripherally to the brain (21,69,70).

Severe CI was also associated with reduced capacity for bacterial biosynthesis of the SCFAs butyrate, acetate, and propionate. SCFA production is known to be important for normal cognitive function and in preventing neuroinflammation (60,71,72). Previous studies have identified an association between a reduced capacity for SCFA biosynthesis and the development of a chronic and systemic inflammatory state, commonly referred to as “inflammaging,” involving increased circulation of IL-6, TNF-α, and C-reactive protein (73–75). Inflammaging, particularly in the brain, is associated with decreased neuronal arborization, numbers of neurons and synapses, and overall brain cortical volume (76) and has been implicated in the acceleration of dementia onset (77,78), and the rate of neurological deterioration (79,80).

In contrast to a decreased capacity for SCFA synthesis, we observed a greater capacity for methanogenesis with increasing CI severity. This relationship was apparent from the representation of methanogenic pathways within the metagenome, and from the increased relative abundance of species, such as *Methanobrevibacter smithii*, in those with severe CI. Increasing capacity for methanogenesis within the gut microbiome has been reported previously in 2 cohorts of centenarians (81,82), as well as in rodent models of aging (83). While the clinical consequences of increased methane production in the gut are poorly understood, high levels are associated with functional constipation (84), diverticulosis (85), and colon cancer (86).

The gut microbiome in participants with more severe CI was found to be depleted in its capacity to synthesize amino acids, particularly L-arginine. The availability of arginine is critical to the regulation of autophagy (87), the cellular process that involves the recycling of nutrients from macromolecules in response to nutrient deficiency (88) and the removal of damaged material from the cellular environment (89). Genetic polymorphisms in genes involved in the regulation of autophagy have been linked to a number of neurodegenerative diseases, including Alzheimer’s, Parkinson’s, Huntington’s, and Lewy body diseases, frontotemporal dementia, and amyotrophic lateral sclerosis (90–94). The conversion of arginine to putrescine, spermidine, and spermine by intestinal microbes promotes autophagy (95–97) and the significant reduction in arginine biosynthesis capacity is consistent with the contribution of suppressed autophagy to the development and progression of age-related disease. Severe CI was also associated with a reduced capacity for microbial production of the essential amino acids, L-valine and L-lysine. Impaired L-valine production has been linked with declining neurological health previously (98), and diet supplementation with L-lysine and L-valine has been shown to improve cognitive and psychological function in older adults (99).

Microbial functions, such as those associated with CI severity, can often be performed by many different members of the gut microbiota. This phenomenon is referred to as functional redundancy and can result in relationships between individual microbial species and host measures of disease being less strong than those based on conserved microbial functional traits. Despite this, we observed a number of discrete bacterial taxa that were significantly associated with CI. In particular, *Methanobrevibacter smithii* and *Alistipes finegoldii* were more prevalent in those with severe CI, while *Bacteroides uniformis* was less highly represented. As above, *Methanobrevibacter smithii* is associated with higher methane production (100) and has been identified as an inflammatory and cardiometabolic biomarker (101). While the precise mechanisms of *Alistipes* species in health and disease are still unclear (102), clinical studies of inflammatory diseases have shown *Alistipes finegoldii* triggers intestinal inflammation and decreases SCFA-producing bacteria, potentially playing a pathogenic role in chronic diseases (102–104).

We also observed severe CI to be associated with a lower prevalence of bacterial taxa that are considered broadly beneficial. These included *Bacteroides uniformis*, which is associated with reduced risk of colorectal cancer (105) and inflammatory bowel disease (106), and *Blautia* species, which have the potential to inhibit the growth of pathogenic bacteria in the intestine and reduce inflammation (107,108). Taxa that have been previously associated with aspects of cognition, such as *Collinsella aerofaciens* (44), were more prevalent in those with severe CI. However, other bacterial taxa associated with aspects of aging, frailty, and cognitive decline in previous studies, including *Faecalibacterium prausnitzii* (59), *Eubacterium rectale* (44,109), and *Escherichia coli* (110–112), were not associated with CI severity in our cohort.

In addition to the relative abundance of different bacterial species, analysis of the broad structure of the gut microbiota can also be informative. We assessed 3 different alpha diversity measures, Shannon–Wiener diversity, Pielou’s evenness, and species richness, that together provide an overview of microbiota structure. While evenness, which represents the relative differences in the abundance of various species in the community, was not associated significantly with CI severity, Shannon–Wiener diversity and species richness were both higher in severe CI. Studies of the gut microbiota in aging have previously reported reduced diversity in older age. For example, Verdi and colleagues identified significantly lower fecal microbiota diversity to be associated with longer reaction times (in cognitive assessments) in an independently living aged cohort (4). Similarly, Wasser and colleagues reported reduced alpha diversity in those with Huntington’s disease (34), while 2 other studies have reported no significant relationship between CI severity and alpha diversity (39,113). In our analysis, where age was adjusted for, a contrary effect was observed, consistent with an association between CI and specific microbiome changes that is separate to wider microbial shifts that are typical in later life.

Our study used the ACFI assessment tool to ascertain CI severity. This tool has been employed by the Australian Commonwealth Government as a basis for care funding for all residents of long-term care facilities across Australia since 2008, is completed by trained assessors, and includes the PAS-CIS (a validated and consistently applied tool of CI in aged care). However, there are limitations to this tool. People may not be comprehensively assessed for CI if they have a sensory/speech impairment, are non-English speaking, or have severe CI beyond the scope of the instrument, which can include a concurrent diagnosis of dementia or mental disorder (47,48). The ACFI is also designed for funding purposes, not clinical care or epidemiological surveillance, which likely results in underreporting of these chronic health conditions (114). Finally, the tool is deployed at entry into aged care and not consistently reevaluated, leading to potential inconsistent time periods in CI assessment. In this study, the mean time between CI assessment and stool collection (16 months), and differences in mean time between groups, is within a period where clinically significant changes to CI are estimated to be minimal (115,116). Nevertheless, time between stool collection and CI assessment was included as a potential confounding variable in all analyses, as has been shown previously (117).

Our study had other limitations that should be considered. First, we were able to relate changes in intestinal microbiology to cognitive function, but not to specific aspects of host neurological pathophysiology. Second, the relationships identified between taxonomic and functional features of the intestinal microbiome and CI are associative and whether they contribute directly to the development and progression of CI remains to be established. Third, the possibility that CI severity drives alterations in microbiome composition, mediated by factors such as dementia medications, changes in food preparation for those with dysphagia, and isolation to locked wards for residents with severe behavioral care needs, cannot be excluded based on the current analysis. Indeed, changes in behavior associated with psychiatric conditions in other contexts, particularly those relating to diet, have been shown to contribute to disease-specific gut microbiome markers (118).

While our analysis involved participants from 5 facilities in metropolitan South Australia, the findings are likely to be representative of a wider phenomenon. Alignment of the GRACE cohort to the comprehensive Registry of Senior Australians (ROSA) (119), a cohort that includes over 2.8 million Australians (including those in long-term aged care), supported the representative nature of our study cohort (119).

## Conclusions

We report age-, sex-, antibiotic-, and diet-independent microbial markers of severe CI. Our analysis implicates multiple gut microbiome–brain pathways in aging-associated cognitive decline, including those involved in inflammation, neurotransmission, and autophagy. These findings raise the possibility of identifying cognitive decline and slowing its rate of progression via microbiome-targeted therapeutic interventions.

## Supplementary Material

glaf053_suppl_Supplementary_Materials

## Data Availability

The GRACE study data are available upon reasonable request. GRACE study data described in this article are available to all interested researchers through collaboration. Please contact G.B.R. (geraint.rogers@sahmri.com). The metagenomic data for this study have been deposited in the European Nucleotide Archive (ENA) at EMBL-EBI under accession number PRJEB51408 (https://www.ebi.ac.uk/ena/browser/view/PRJEB51408). The metabolomic data for this study are available at the NIH Common Fund’s National Metabolomics Data Repository (NMDR) website, the Metabolomics Workbench, https://www.metabolomicsworkbench.org (120), where it has been assigned Project ID PR001631. The metabolomic data can also be accessed directly via its Project DOI: http://dx.doi.org/10.21228/M8W43G. Regarding the sharing of linked data from ROSA, due to data custodian restrictions, individual ROSA data cannot be made publicly available to other researchers.

## References

[1] Albert MS, Jones K, Savage CR, et al Predictors of cognitive change in older persons: MacArthur Studies of Successful Aging. Psychol Aging. 1995;10(4):578–589. https://doi.org/10.1037//0882-7974.10.4.578 8749585

[2] Comijs HC, Dik MG, Deeg DJ, et al The course of cognitive decline in older persons: Results from the Longitudinal Aging Study Amsterdam. Dement Geriatr Cogn Disord. 2004;17(3):136–142. https://doi.org/10.1159/000076346 14739534

[3] Harada CN, Natelson Love MC, Triebel KL. Normal cognitive aging. Clin Geriatr Med. 2013;29(4):737–752. https://doi.org/10.1016/j.cger.2013.07.002 24094294 PMC4015335

[4] Verdi S, Jackson MA, Beaumont M, et al An investigation into physical frailty as a link between the gut microbiome and cognitive health. Front Aging Neurosci. 2018;10:398. https://doi.org/10.3389/fnagi.2018.00398 30564113 PMC6288358

[5] Henon H, Durieu I, Guerouaou D, et al Poststroke dementia: Incidence and relationship to prestroke cognitive decline. Neurology. 2001;57(7):1216–1222. https://doi.org/10.1212/wnl.57.7.1216 11591838

[6] Ye BS, Seo SW, Kim JH, et al Effects of amyloid and vascular markers on cognitive decline in subcortical vascular dementia. Neurology. 2015;85(19):1687–1693. https://doi.org/10.1212/WNL.0000000000002097 26468407 PMC4653105

[7] Bussian TJ, Aziz A, Meyer CF, et al Clearance of senescent glial cells prevents tau-dependent pathology and cognitive decline. Nature. 2018;562(7728):578–582. https://doi.org/10.1038/s41586-018-0543-y 30232451 PMC6206507

[8] Luo R, Su LY, Li G, et al Activation of PPARA-mediated autophagy reduces Alzheimer disease-like pathology and cognitive decline in a murine model. Autophagy. 2020;16(1):52–69. https://doi.org/10.1080/15548627.2019.1596488 30898012 PMC6984507

[9] Lin CH, Yang SY, Horng HE, et al Plasma alpha-synuclein predicts cognitive decline in Parkinson’s disease. J Neurol Neurosurg Psychiatry. 2017;88(10):818–824. https://doi.org/10.1136/jnnp-2016-314857 28550072 PMC5629933

[10] Mattila PM, Rinne JO, Helenius H, et al Alpha-synuclein-immunoreactive cortical Lewy bodies are associated with cognitive impairment in Parkinson’s disease. Acta Neuropathol. 2000;100(3):285–290. https://doi.org/10.1007/s004019900168 10965798

[11] Koistinaho M, Lin S, Wu X, et al Apolipoprotein E promotes astrocyte colocalization and degradation of deposited amyloid-beta peptides. Nat Med. 2004;10(7):719–726. https://doi.org/10.1038/nm1058 15195085

[12] Lin YT, Seo J, Gao F, et al APOE4 causes widespread molecular and cellular alterations associated with Alzheimer’s disease phenotypes in human iPSC-derived brain cell types. Neuron. 2018;98(6):1141–1154.e7. https://doi.org/10.1016/j.neuron.2018.05.008 29861287 PMC6023751

[13] Liu CC, Zhao N, Fu Y, et al ApoE4 accelerates early seeding of amyloid pathology. Neuron. 2017;96(5):1024–1032.e3. https://doi.org/10.1016/j.neuron.2017.11.013 29216449 PMC5948105

[14] Parcon PA, Balasubramaniam M, Ayyadevara S, et al Apolipoprotein E4 inhibits autophagy gene products through direct, specific binding to CLEAR motifs. Alzheimers Dement. 2018;14(2):230–242. https://doi.org/10.1016/j.jalz.2017.07.754 28945989 PMC6613789

[15] Shi Y, Yamada K, Liddelow SA, et al; Alzheimer’s Disease Neuroimaging Initiative. ApoE4 markedly exacerbates tau-mediated neurodegeneration in a mouse model of tauopathy. Nature. 2017;549(7673):523–527. https://doi.org/10.1038/nature24016 28959956 PMC5641217

[16] Ulrich JD, Ulland TK, Mahan TE, et al ApoE facilitates the microglial response to amyloid plaque pathology. J Exp Med. 2018;215(4):1047–1058. https://doi.org/10.1084/jem.20171265 29483128 PMC5881464

[17] Livingston G, Huntley J, Sommerlad A, et al Dementia prevention, intervention, and care: 2020 report of the Lancet Commission. Lancet. 2020;396(10248):413–446. https://doi.org/10.1016/S0140-6736(20)30367-6 32738937 PMC7392084

[18] Cryan JF, O’Riordan KJ, Cowan CSM, et al The microbiota-gut-brain axis. Physiol Rev. 2019;99(4):1877–2013. https://doi.org/10.1152/physrev.00018.2018 31460832

[19] Rogers GB, Keating DJ, Young RL, et al From gut dysbiosis to altered brain function and mental illness: Mechanisms and pathways. Mol Psychiatry. 2016;21(6):738–748. https://doi.org/10.1038/mp.2016.50 27090305 PMC4879184

[20] Sharon G, Sampson TR, Geschwind DH, et al The central nervous system and the gut microbiome. Cell. 2016;167(4):915–932. https://doi.org/10.1016/j.cell.2016.10.027 27814521 PMC5127403

[21] Shoubridge AP, Choo JM, Martin AM, et al The gut microbiome and mental health: Advances in research and emerging priorities. Mol Psychiatry. 2022;27(4):1908–1919. https://doi.org/10.1038/s41380-022-01479-w 35236957

[22] Valles-Colomer M, Falony G, Darzi Y, et al The neuroactive potential of the human gut microbiota in quality of life and depression. Nat Microbiol. 2019;4(4):623–632. https://doi.org/10.1038/s41564-018-0337-x 30718848

[23] Yano JM, Yu K, Donaldson GP, et al Indigenous bacteria from the gut microbiota regulate host serotonin biosynthesis. Cell. 2015;161(2):264–276. https://doi.org/10.1016/j.cell.2015.02.047 25860609 PMC4393509

[24] Correa-Oliveira R, Fachi JL, Vieira A, et al Regulation of immune cell function by short-chain fatty acids. Clin Transl Immunology. 2016;5(4):e73. https://doi.org/10.1038/cti.2016.17 27195116 PMC4855267

[25] Dalile B, Van Oudenhove L, Vervliet B, et al The role of short-chain fatty acids in microbiota-gut-brain communication. Nat Rev Gastroenterol Hepatol. 2019;16(8):461–478. https://doi.org/10.1038/s41575-019-0157-3 31123355

[26] Lai WT, Zhao J, Xu SX, et al Shotgun metagenomics reveals both taxonomic and tryptophan pathway differences of gut microbiota in bipolar disorder with current major depressive episode patients. J Affect Disord. 2021;278:311–319. https://doi.org/10.1016/j.jad.2020.09.010 32979562

[27] Marx W, McGuinness AJ, Rocks T, et al The kynurenine pathway in major depressive disorder, bipolar disorder, and schizophrenia: A meta-analysis of 101 studies. Mol Psychiatry. 2021;26(8):4158–4178. https://doi.org/10.1038/s41380-020-00951-9 33230205

[28] Bonfili L, Cecarini V, Gogoi O, et al Microbiota modulation as preventative and therapeutic approach in Alzheimer’s disease. FEBS J. 2021;288(9):2836–2855. https://doi.org/10.1111/febs.15571 32969566

[29] Soto M, Herzog C, Pacheco JA, et al Gut microbiota modulate neurobehavior through changes in brain insulin sensitivity and metabolism. Mol Psychiatry. 2018;23(12):2287–2301. https://doi.org/10.1038/s41380-018-0086-5 29910467 PMC6294739

[30] Kim MS, Kim Y, Choi H, et al Transfer of a healthy microbiota reduces amyloid and tau pathology in an Alzheimer’s disease animal model. Gut. 2020;69(2):283–294. https://doi.org/10.1136/gutjnl-2018-317431 31471351

[31] Vogt NM, Kerby RL, Dill-McFarland KA, et al Gut microbiome alterations in Alzheimer’s disease. Sci Rep. 2017;7(1):13537. https://doi.org/10.1038/s41598-017-13601-y 29051531 PMC5648830

[32] Bjorkqvist M, Wild EJ, Thiele J, et al A novel pathogenic pathway of immune activation detectable before clinical onset in Huntington’s disease. J Exp Med. 2008;205(8):1869–1877. https://doi.org/10.1084/jem.20080178 18625748 PMC2525598

[33] Du G, Dong W, Yang Q, et al Altered gut microbiota related to inflammatory responses in patients with Huntington’s disease. Front Immunol. 2020;11:603594. https://doi.org/10.3389/fimmu.2020.603594 33679692 PMC7933529

[34] Wasser CI, Mercieca EC, Kong G, et al Gut dysbiosis in Huntington’s disease: Associations among gut microbiota, cognitive performance and clinical outcomes. Brain Commun. 2020;2(2):fcaa110. https://doi.org/10.1093/braincomms/fcaa110 33005892 PMC7519724

[35] Sampson TR, Debelius JW, Thron T, et al Gut microbiota regulate motor deficits and neuroinflammation in a model of Parkinson’s disease. Cell. 2016;167(6):1469–1480.e12. https://doi.org/10.1016/j.cell.2016.11.018 27912057 PMC5718049

[36] Sun MF, Zhu YL, Zhou ZL, et al Neuroprotective effects of fecal microbiota transplantation on MPTP-induced Parkinson’s disease mice: Gut microbiota, glial reaction and TLR4/TNF-alpha signaling pathway. Brain Behav Immun. 2018;70:48–60. https://doi.org/10.1016/j.bbi.2018.02.005 29471030

[37] Claesson MJ, Cusack S, O’Sullivan O, et al Composition, variability, and temporal stability of the intestinal microbiota of the elderly. Proc Natl Acad Sci USA. 2011;108(Suppl 1):4586–4591. https://doi.org/10.1073/pnas.1000097107 20571116 PMC3063589

[38] Meyer K, Lulla A, Debroy K, et al Association of the gut microbiota with cognitive function in midlife. JAMA Netw Open. 2022;5(2):e2143941. https://doi.org/10.1001/jamanetworkopen.2021.43941 35133436 PMC8826173

[39] Komanduri M, Savage K, Lea A, et al The relationship between gut microbiome and cognition in older Australians. Nutrients. 2021;14(1):64. https://doi.org/10.3390/nu14010064 35010939 PMC8746300

[40] Manderino L, Carroll I, Azcarate-Peril MA, et al Preliminary evidence for an association between the composition of the gut microbiome and cognitive function in neurologically healthy older adults. J Int Neuropsychol Soc. 2017;23(8):700–705. https://doi.org/10.1017/S1355617717000492 28641593 PMC6111127

[41] Desbonnet L, Clarke G, Traplin A, et al Gut microbiota depletion from early adolescence in mice: implications for brain and behaviour. Brain Behav Immun. 2015;48:165–173. https://doi.org/10.1016/j.bbi.2015.04.004 25866195

[42] Frohlich EE, Farzi A, Mayerhofer R, et al Cognitive impairment by antibiotic-induced gut dysbiosis: Analysis of gut microbiota-brain communication. Brain Behav Immun. 2016;56:140–155. https://doi.org/10.1016/j.bbi.2016.02.020 26923630 PMC5014122

[43] Lynn MA, Eden G, Ryan FJ, et al The composition of the gut microbiota following early-life antibiotic exposure affects host health and longevity in later life. Cell Rep. 2021;36(8):109564. https://doi.org/10.1016/j.celrep.2021.109564 34433065

[44] Ghosh TS, Rampelli S, Jeffery IB, et al Mediterranean diet intervention alters the gut microbiome in older people reducing frailty and improving health status: The NU-AGE 1-year dietary intervention across five European countries. Gut. 2020;69(7):1218–1228. https://doi.org/10.1136/gutjnl-2019-319654 32066625 PMC7306987

[45] D’Amato A, Di Cesare Mannelli L, Lucarini E, et al Faecal microbiota transplant from aged donor mice affects spatial learning and memory via modulating hippocampal synaptic plasticity- and neurotransmission-related proteins in young recipients. Microbiome. 2020;8(1):140. https://doi.org/10.1186/s40168-020-00914-w 33004079 PMC7532115

[46] Carpenter L, Shoubridge A, Flynn E, et al; on behalf of the GRACE Investigative Study Team. Generating *e*vidence on Resistant *b*acteria in the Aged Care Environment (GRACE) 2021 Report. 2021:47. https://www.gracestudy.com.au/post/grace-study-stage-2-report. Accessed 15 April, 2024.

[47] Department of Health and Ageing. Aged *Care Funding Instrument* (ACFI) *User Guide*. Department of Health and Ageing; 2016:48.

[48] Australian Institute of Health and Welfare (AIHW). Aged Care Assessment Program Data Dictionary. AIHW; 2002.

[49] Jorm AF, Mackinnon AJ, Henderson AS, et al The Psychogeriatric Assessment Scales: A multi-dimensional alternative to categorical diagnoses of dementia and depression in the elderly. Psychol Med. 1995;25(3):447–460. https://doi.org/10.1017/s0033291700033377 7480426

[50] Motulsky HJ, Brown RE. Detecting outliers when fitting data with nonlinear regression—a new method based on robust nonlinear regression and the false discovery rate. BMC Bioinf. 2006;7:123. https://doi.org/10.1186/1471-2105-7-123 PMC1472692 16526949

[51] Bolger AM, Lohse M, Usadel B. Trimmomatic: A flexible trimmer for Illumina sequence data. Bioinformatics. 2014;30(15):2114–2120. https://doi.org/10.1093/bioinformatics/btu170 24695404 PMC4103590

[52] Langmead B, Salzberg SL. Fast gapped-read alignment with Bowtie 2. Nat Methods. 2012;9(4):357–359. https://doi.org/10.1038/nmeth.1923 22388286 PMC3322381

[53] Beghini F, McIver LJ, Blanco-Miguez A, et al Integrating taxonomic, functional, and strain-level profiling of diverse microbial communities with bioBakery 3. Elife. 2021;10:e65088. https://doi.org/10.7554/eLife.65088 33944776 PMC8096432

[54] Peet RK. The measurement of species diversity. Annu Rev Ecol Syst. 1974;5(1):285–307. https://doi.org/10.1146/annurev.es.05.110174.001441

[55] Oksanen J, Simpson GL, Blanchet FG, et al vegan: Community Ecology Package. In R package version 2.6-2. 2022. https://CRAN.R-project.org/package=vegan. Accessed 15 April, 2024.

[56] Lozupone C, Knight R. UniFrac: A new phylogenetic method for comparing microbial communities. Appl Environ Microbiol. 2005;71(12):8228–8235. https://doi.org/10.1128/AEM.71.12.8228-8235.2005 16332807 PMC1317376

[57] Gubert C, Choo JM, Love CJ, et al Faecal microbiota transplant ameliorates gut dysbiosis and cognitive deficits in Huntington’s disease mice. Brain Commun. 2022;4(4):fcac205. https://doi.org/10.1093/braincomms/fcac205 36035436 PMC9400176

[58] Kong G, Ellul S, Narayana VK, et al An integrated metagenomics and metabolomics approach implicates the microbiota-gut-brain axis in the pathogenesis of Huntington’s disease. Neurobiol Dis. 2021;148:105199. https://doi.org/10.1016/j.nbd.2020.105199 33249136

[59] Jackson MA, Jeffery IB, Beaumont M, et al Signatures of early frailty in the gut microbiota. Genome Med. 2016;8(1):8. https://doi.org/10.1186/s13073-016-0262-7 26822992 PMC4731918

[60] Erny D, Hrabe de Angelis AL, Jaitin D, et al Host microbiota constantly control maturation and function of microglia in the CNS. Nat Neurosci. 2015;18(7):965–977. https://doi.org/10.1038/nn.4030 26030851 PMC5528863

[61] Bellono NW, Bayrer JR, Leitch DB, et al Enterochromaffin cells are gut chemosensors that couple to sensory neural pathways. Cell. 2017;170(1):185–198.e16. https://doi.org/10.1016/j.cell.2017.05.034 28648659 PMC5839326

[62] Ye L, Bae M, Cassilly CD, et al Enteroendocrine cells sense bacterial tryptophan catabolites to activate enteric and vagal neuronal pathways. Cell Host Microbe. 2021;29(2):179–196.e9. https://doi.org/10.1016/j.chom.2020.11.011 33352109 PMC7997396

[63] Arentsen T, Qian Y, Gkotzis S, et al The bacterial peptidoglycan-sensing molecule Pglyrp2 modulates brain development and behavior. Mol Psychiatry. 2017;22(2):257–266. https://doi.org/10.1038/mp.2016.182 27843150 PMC5285465

[64] Kim MH, Kang SG, Park JH, et al Short-chain fatty acids activate GPR41 and GPR43 on intestinal epithelial cells to promote inflammatory responses in mice. Gastroenterology. 2013;145(2):396–406.e1. https://doi.org/10.1053/j.gastro.2013.04.056 23665276

[65] Gueli MC, Taibi G. Alzheimer’s disease: Amino acid levels and brain metabolic status. Neurol Sci. 2013;34(9):1575–1579. https://doi.org/10.1007/s10072-013-1289-9 23354600

[66] Jimenez-Jimenez FJ, Molina JA, Gomez P, et al Neurotransmitter amino acids in cerebrospinal fluid of patients with Alzheimer’s disease. J Neural Transm (Vienna). 1998;105(2-3):269–277. https://doi.org/10.1007/s007020050056 9660105

[67] Lin CH, Yang HT, Lane HY. D-glutamate, D-serine, and D-alanine differ in their roles in cognitive decline in patients with Alzheimer’s disease or mild cognitive impairment. Pharmacol Biochem Behav. 2019;185:172760. https://doi.org/10.1016/j.pbb.2019.172760 31422081

[68] Murley AG, Rouse MA, Jones PS, et al GABA and glutamate deficits from frontotemporal lobar degeneration are associated with disinhibition. Brain. 2020;143(11):3449–3462. https://doi.org/10.1093/brain/awaa305 33141154 PMC7719029

[69] Kaelberer MM, Buchanan KL, Klein ME, et al A gut-brain neural circuit for nutrient sensory transduction. Science. 2018;361(6408):eaat5236. https://doi.org/10.1126/science.aat5236 30237325 PMC6417812

[70] Strandwitz P. Neurotransmitter modulation by the gut microbiota. Brain Res. 2018;1693(Pt B):128–133. https://doi.org/10.1016/j.brainres.2018.03.015 29903615 PMC6005194

[71] Arnoldussen IAC, Wiesmann M, Pelgrim CE, et al Butyrate restores HFD-induced adaptations in brain function and metabolism in mid-adult obese mice. Int J Obes (Lond). 2017;41(6):935–944. https://doi.org/10.1038/ijo.2017.52 28220041

[72] Byrne CS, Chambers ES, Alhabeeb H, et al Increased colonic propionate reduces anticipatory reward responses in the human striatum to high-energy foods. Am J Clin Nutr. 2016;104(1):5–14. https://doi.org/10.3945/ajcn.115.126706 27169834 PMC4919527

[73] Franceschi C, Campisi J. Chronic inflammation (inflammaging) and its potential contribution to age-associated diseases. J Gerontol A Biol Sci Med Sci. 2014;69(Suppl 1):S4–S9. https://doi.org/10.1093/gerona/glu057 24833586

[74] Franceschi C, Garagnani P, Parini P, et al Inflammaging: A new immune-metabolic viewpoint for age-related diseases. Nat Rev Endocrinol. 2018;14(10):576–590. https://doi.org/10.1038/s41574-018-0059-4 30046148

[75] Frasca D, Blomberg BB. Inflammaging decreases adaptive and innate immune responses in mice and humans. Biogerontology. 2016;17(1):7–19. https://doi.org/10.1007/s10522-015-9578-8 25921609 PMC4626429

[76] Stephenson J, Nutma E, van der Valk P, Amor S. Inflammation in CNS neurodegenerative diseases. Immunology. 2018;154(2):204–219. https://doi.org/10.1111/imm.12922 29513402 PMC5980185

[77] Grande G, Marengoni A, Vetrano DL, et al Multimorbidity burden and dementia risk in older adults: The role of inflammation and genetics. Alzheimers Dement. 2021;17(5):768–776. https://doi.org/10.1002/alz.12237 33403740 PMC8247430

[78] Sankowski R, Mader S, Valdes-Ferrer SI. Systemic inflammation and the brain: Novel roles of genetic, molecular, and environmental cues as drivers of neurodegeneration. Front Cell Neurosci. 2015;9:28. https://doi.org/10.3389/fncel.2015.00028 25698933 PMC4313590

[79] Eikelenboom P, Hoozemans JJ, Veerhuis R, et al Whether, when and how chronic inflammation increases the risk of developing late-onset Alzheimer’s disease. Alzheimers Res Ther. 2012;4(3):15. https://doi.org/10.1186/alzrt118 22647384 PMC3506930

[80] Giunta B, Fernandez F, Nikolic WV, et al Inflammaging as a prodrome to Alzheimer’s disease. J Neuroinflammation. 2008;5:51. https://doi.org/10.1186/1742-2094-5-51 19014446 PMC2615427

[81] Li C, Luan Z, Zhao Y, et al Deep insights into the gut microbial community of extreme longevity in south Chinese centenarians by ultra-deep metagenomics and large-scale culturomics. NPJ Biofilms Microbiomes. 2022;8(1):28. https://doi.org/10.1038/s41522-022-00282-3 35440640 PMC9019030

[82] Wu L, Zeng T, Zinellu A, et al A cross-sectional study of compositional and functional profiles of gut microbiota in sardinian centenarians. mSystems. 2019;4(4):e00325–19. https://doi.org/10.1128/mSystems.00325-19 31289141 PMC6616150

[83] Maczulak AE, Wolin MJ, Miller TL. Increase in colonic methanogens and total anaerobes in aging rats. Appl Environ Microbiol. 1989;55(10):2468–2473. https://doi.org/10.1128/aem.55.10.2468-2473.1989 2604389 PMC203106

[84] Chatterjee S, Park S, Low K, et al The degree of breath methane production in IBS correlates with the severity of constipation. Am J Gastroenterol. 2007;102(4):837–841. https://doi.org/10.1111/j.1572-0241.2007.01072.x 17397408

[85] Weaver GA, Krause JA, Miller TL, et al Incidence of methanogenic bacteria in a sigmoidoscopy population: An association of methanogenic bacteria and diverticulosis. Gut. 1986;27(6):698–704. https://doi.org/10.1136/gut.27.6.698 3721294 PMC1433329

[86] Haines A, Metz G, Dilawari J, et al Breath-methane in patients with cancer of the large bowel. Lancet. 1977;2(8036):481–483. https://doi.org/10.1016/s0140-6736(77)91605-1 70691

[87] Poillet-Perez L, Xie X, Zhan L, et al Autophagy maintains tumour growth through circulating arginine. Nature. 2018;563(7732):569–573. https://doi.org/10.1038/s41586-018-0697-7 30429607 PMC6287937

[88] Mizushima N. Methods for monitoring autophagy. Int J Biochem Cell Biol. 2004;36(12):2491–2502. https://doi.org/10.1016/j.biocel.2004.02.005 15325587

[89] Lazarou M, Sliter DA, Kane LA, et al The ubiquitin kinase PINK1 recruits autophagy receptors to induce mitophagy. Nature. 2015;524(7565):309–314. https://doi.org/10.1038/nature14893 26266977 PMC5018156

[90] Fujikake N, Shin M, Shimizu S. Association between autophagy and neurodegenerative diseases. Front Neurosci. 2018;12:255. https://doi.org/10.3389/fnins.2018.00255 29872373 PMC5972210

[91] Gan L, Cookson MR, Petrucelli L, et al Converging pathways in neurodegeneration, from genetics to mechanisms. Nat Neurosci. 2018;21(10):1300–1309. https://doi.org/10.1038/s41593-018-0237-7 30258237 PMC6278826

[92] Gao S, Casey AE, Sargeant TJ, et al Genetic variation within endolysosomal system is associated with late-onset Alzheimer’s disease. Brain. 2018;141(9):2711–2720. https://doi.org/10.1093/brain/awy197 30124770

[93] Nixon RA. The role of autophagy in neurodegenerative disease. Nat Med. 2013;19(8):983–997. https://doi.org/10.1038/nm.3232 23921753

[94] Tsuang D, Leverenz JB, Lopez OL, et al GBA mutations increase risk for Lewy body disease with and without Alzheimer disease pathology. Neurology. 2012;79(19):1944–1950. https://doi.org/10.1212/WNL.0b013e3182735e9a 23035075 PMC3484986

[95] Eisenberg T, Knauer H, Schauer A, et al Induction of autophagy by spermidine promotes longevity. Nat Cell Biol. 2009;11(11):1305–1314. https://doi.org/10.1038/ncb1975 19801973

[96] Oliphant K, Allen-Vercoe E. Macronutrient metabolism by the human gut microbiome: Major fermentation by-products and their impact on host health. Microbiome. 2019;7(1):91. https://doi.org/10.1186/s40168-019-0704-8 31196177 PMC6567490

[97] Pugin B, Barcik W, Westermann P, et al A wide diversity of bacteria from the human gut produces and degrades biogenic amines. Microb Ecol Health Dis. 2017;28(1):1353881. https://doi.org/10.1080/16512235.2017.1353881 28959180 PMC5614385

[98] Baranyi A, Amouzadeh-Ghadikolai O, von Lewinski D, et al Branched-chain amino acids as new biomarkers of major depression—a novel neurobiology of mood disorder. PLoS One. 2016;11(8):e0160542. https://doi.org/10.1371/journal.pone.0160542 27490818 PMC4973973

[99] Suzuki H, Yamashiro D, Ogawa S, et al Intake of seven essential amino acids improves cognitive function and psychological and social function in middle-aged and older adults: A double-blind, randomized, placebo-controlled trial. Front Nutr. 2020;7:586166. https://doi.org/10.3389/fnut.2020.586166 33324669 PMC7724102

[100] Ghoshal U, Shukla R, Srivastava D, et al Irritable bowel syndrome, particularly the constipation-predominant form, involves an increase in *Methanobrevibacter smithii*, which is associated with higher methane production. Gut Liver. 2016;10(6):932–938. https://doi.org/10.5009/gnl15588 27458176 PMC5087933

[101] Fu BC, Hullar MAJ, Randolph TW, et al Associations of plasma trimethylamine N-oxide, choline, carnitine, and betaine with inflammatory and cardiometabolic risk biomarkers and the fecal microbiome in the Multiethnic Cohort Adiposity Phenotype Study. Am J Clin Nutr. 2020;111(6):1226–1234. https://doi.org/10.1093/ajcn/nqaa015 32055828 PMC7266689

[102] Parker BJ, Wearsch PA, Veloo ACM, et al The genus *Alistipes*: Gut bacteria with emerging implications to inflammation, cancer, and mental health. Front Immunol. 2020;11:906. https://doi.org/10.3389/fimmu.2020.00906 32582143 PMC7296073

[103] Kim S, Goel R, Kumar A, et al Imbalance of gut microbiome and intestinal epithelial barrier dysfunction in patients with high blood pressure. Clin Sci (Lond). 2018;132(6):701–718. https://doi.org/10.1042/CS20180087 29507058 PMC5955695

[104] Rodriguez-Palacios A, Conger M, Hopperton A, et al Identification of pathogenic bacteria in severe Crohn’s disease. Inflamm Bowel Dis. 2019;25(Supplement_1):SS70–SS70. https://doi.org/10.1093/ibd/izy393.175

[105] Wang T, Cai G, Qiu Y, et al Structural segregation of gut microbiota between colorectal cancer patients and healthy volunteers. ISME J. 2012;6(2):320–329. https://doi.org/10.1038/ismej.2011.109 21850056 PMC3260502

[106] Takahashi K, Nishida A, Fujimoto T, et al Reduced abundance of butyrate-producing bacteria species in the fecal microbial community in Crohn’s disease. Digestion. 2016;93(1):59–65. https://doi.org/10.1159/000441768 26789999

[107] Hosomi K, Saito M, Park J, et al Oral administration of *Blautia wexlerae* ameliorates obesity and type 2 diabetes via metabolic remodeling of the gut microbiota. Nat Commun. 2022;13(1):4477. https://doi.org/10.1038/s41467-022-32015-7 35982037 PMC9388534

[108] Liu X, Mao B, Gu J, et al *Blautia*—a new functional genus with potential probiotic properties? Gut Microbes. 2021;13(1):1–21. https://doi.org/10.1080/19490976.2021.1875796 PMC7872077 33525961

[109] van Soest APM, Hermes GDA, Berendsen AAM, et al Associations between pro- and anti-inflammatory gastro-intestinal microbiota, diet, and cognitive functioning in Dutch healthy older adults: The NU-Age Study. Nutrients. 2020;12(11):3471. https://doi.org/10.3390/nu12113471 33198235 PMC7697493

[110] Barrientos RM, Watkins LR, Rudy JW, et al Characterization of the sickness response in young and aging rats following *E. coli* infection. Brain Behav Immun. 2009;23(4):450–454. https://doi.org/10.1016/j.bbi.2009.01.016 19486645 PMC2783183

[111] d’Avila JC, Siqueira LD, Mazeraud A, et al Age-related cognitive impairment is associated with long-term neuroinflammation and oxidative stress in a mouse model of episodic systemic inflammation. J Neuroinflammation. 2018;15(1):28. https://doi.org/10.1186/s12974-018-1059-y 29382344 PMC5791311

[112] Hoogland ICM, Westhoff D, Engelen-Lee JY, et al Microglial activation after systemic stimulation with lipopolysaccharide and *Escherichia coli*. Front Cell Neurosci. 2018;12:110. https://doi.org/10.3389/fncel.2018.00110 29755322 PMC5932388

[113] Stadlbauer V, Engertsberger L, Komarova I, et al Dysbiosis, gut barrier dysfunction and inflammation in dementia: A pilot study. BMC Geriatr. 2020;20(1):248. https://doi.org/10.1186/s12877-020-01644-2 32690030 PMC7372911

[114] Lind KE, Raban MZ, Brett L, et al Measuring the prevalence of 60 health conditions in older Australians in residential aged care with electronic health records: A retrospective dynamic cohort study. Popul Health Metr. 2020;18(1):25. https://doi.org/10.1186/s12963-020-00234-z 33032628 PMC7545887

[115] Morris JN, Howard EP, Schachter E, et al Cognitive change among nursing home residents: CogRisk-NH scale development to predict decline. J Am Med Dir Assoc. 2023;24(9):1405–1411. https://doi.org/10.1016/j.jamda.2023.06.011 37517808

[116] Zaninotto P, Batty GD, Allerhand M, et al Cognitive function trajectories and their determinants in older people: 8 years of follow-up in the English Longitudinal Study of Ageing. J Epidemiol Community Health. 2018;72(8):685–694. https://doi.org/10.1136/jech-2017-210116 29691286 PMC6204948

[117] Ferreiro AL, Choi J, Ryou J, et al Gut microbiome composition may be an indicator of preclinical Alzheimer’s disease. Sci Transl Med. 2023;15(700):eabo2984. https://doi.org/10.1126/scitranslmed.abo2984 37315112 PMC10680783

[118] Larroya A, Pantoja J, Codoner-Franch P, et al Towards tailored gut microbiome-based and dietary interventions for promoting the development and maintenance of a healthy brain. Front Pediatr. 2021;9:705859. https://doi.org/10.3389/fped.2021.705859 34277527 PMC8280474

[119] Carpenter L, Shoubridge AP, Flynn E, et al Cohort profile: GRACE—a residential aged care cohort examining factors influencing antimicrobial resistance carriage. BMC Geriatr. 2023;23(1):521. https://doi.org/10.1186/s12877-023-04215-3 37641010 PMC10464000

[120] Sud M, Fahy E, Cotter D, et al Metabolomics workbench: An international repository for metabolomics data and metadata, metabolite standards, protocols, tutorials and training, and analysis tools. Nucleic Acids Res. 2016;44(D1):DD463–DD470. https://doi.org/10.1093/nar/gkv1042 PMC4702780 26467476

