# Supplementary material for: Severe Cognitive Decline in Long-term Care Is Related to Gut Microbiome Production of Metabolites Involved in Neurotransmission, Immunomodulation, and Autophagy
Source: J Gerontol A Biol Sci Med Sci. 2025 Mar 28;80(7):glaf053. doi: 10.1093/gerona/glaf053 (PMC12168445; doi:10.1093/gerona/glaf053)
Supplement: glaf053_suppl_Supplementary_Materials [file glaf053_suppl_supplementary_materials.pdf]

# Severe cognitive decline in long-term care is related to gut microbiome production of metabolites involved in neurotransmission, immunomodulation, and autophagy

Andrew P. Shoubridge[1,2], Lucy Carpenter[1,2], Erin Flynn[1], Lito E. Papanicolas[1,2,3], Josephine Collins[1], David Gordon[3,4], David J. Lynn[2,5], Craig Whitehead[6,7], Lex E.X. Leong[3], Monica Cations[7,8], David P. De Souza[9], Vinod K. Narayana[9], Jocelyn M. Choo[1,2], Steve L. Wesselingh[1,2,7], Maria Crotty[6,7], Maria C. Inacio[7,10], Kerry Ivey[1,11], Steven L. Taylor[1,2#], Geraint B. Rogers[1,2#*]

## SUPPLEMENTARY MATERIAL

## Supplementary Text

## eMethods

**Metabolite profiling methodology**

SCFA analysis was performed using an Agilent 6490 series triple quadrupole mass spectrometer (Agilent Technologies) with chromatographic separation on an Agilent 1200 series high-performance liquid chromatography system (Agilent Technologies). SCFAs were extracted by adding 360 µL of 50% acetonitrile with 10µM 4-methylvaleric acid internal standard to 40 µL of biological sample supernatant. Samples were then vortexed for 30 seconds, incubated at 10°C for 30 minutes at 950 rpm, centrifuged at 14,000 rpm for five minutes at 4°C, followed by supernatant collection. Derivatisation for SCFA analysis was performed by first adding 20 µL of 20 µM $^{13}C_6$-nitrophenylhydrazine as internal standard to 40 µL of the extracted supernatant, followed by 20 µL each of 200 mM nitrophenylhydrazine and 120 mM 1-ethyl-3-(3-dimethylaminopropyl)carbodiimide (EDC), incubated at 40°C for 30 minutes at 950 rpm, quenched with 20 µL of 200 mM quinic acid, and incubated at 40°C for a further 30 minutes at 950 rpm. Lastly, the samples were reconstituted with 1.9 mL of 15% acetonitrile and 1 µL was injected onto the column. Pooled biological quality controls (PBQCs) were created by pooling extracts (20 µL) from individual biological samples and injected onto the column in five sample intervals. A reagent and procedural blank of the original sample preservation buffer was included for analysis to perform background correction.

Polar metabolite analysis was performed using an Agilent 6545 series quadrupole time-of-flight mass spectrometer (Agilent Technologies) with chromatographic separation on an Agilent 1200 series HPLC system (Agilent Technologies). Metabolite extraction was performed by first adding a solvent mixture of acetonitrile, methanol and water to 20 µL of biological sample, followed by vortexing, sonication, and agitation. Samples were centrifuged and supernatant collected and mixed with an internal standard mixture containing $^{13}C_5$, $^{15}N$-valine, $^{13}C_6$-leucine, and $^{13}C_6$-sorbitol, and 14 µL of sample was injected onto the column. Samples were injected in a randomised order and PBQCs were injected onto the column in five sample intervals.

## Supplementary Tables and Figures

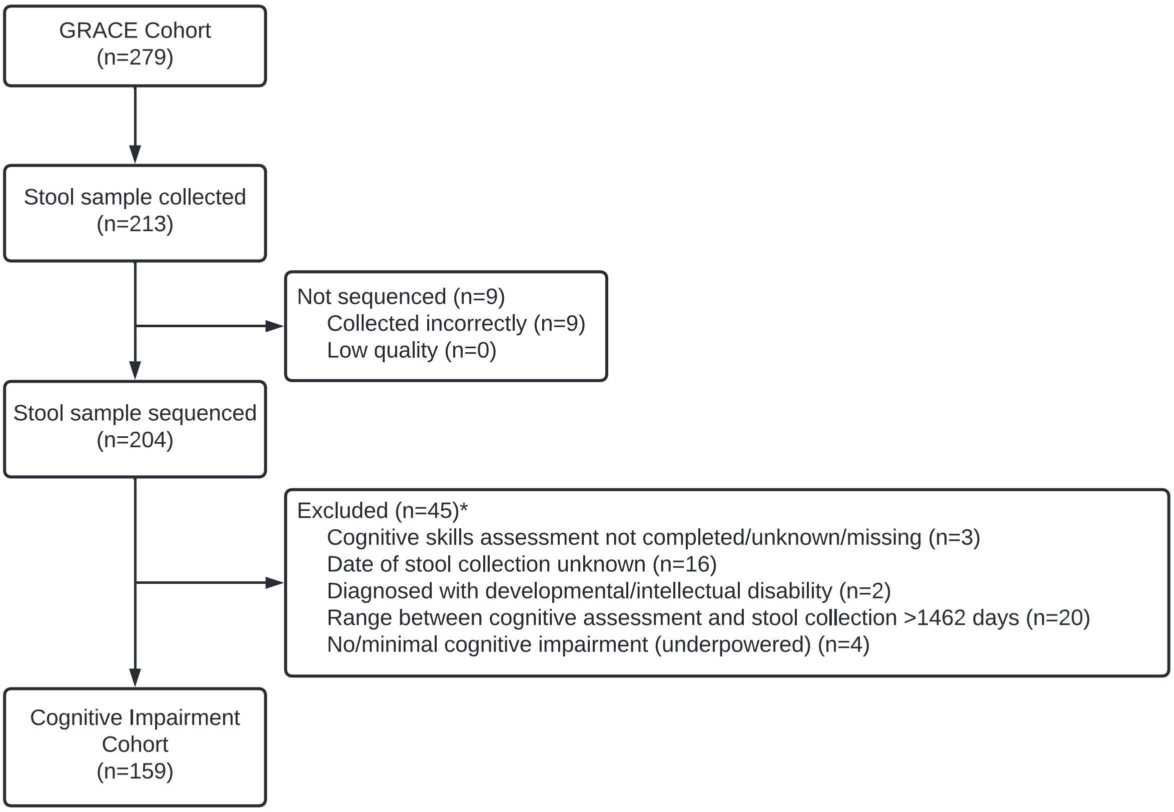


**eFigure 1. Selection of the Cognitive Impairment cohort.**
Inclusion and exclusion numbers for the Cognitive Impairment cohort derived from the original GRACE cohort.
*Total number of participants excluded that met one or more of the exclusion criteria.

## eTable 1. Cognitive Impairment cohort characteristics compared with the GRACE cohort.

| Demographic | GRACE cohort (n=279) | Cognitive Impairment cohort (n=159) |
|---|---|---|
| **Age (years):** | | |
| Median (IQR) | 88.6 (81.8,93.2) | 88.6 (82.0,93.5) |
| **Sex:** % (n) | | |
| Female | 71.7 (200) | 67.7 (109) |
| Male | 28.3 (79) | 32.3 (52) |
| **Time lived in facility (days):** | | |
| Median (IQR) | 681 (252,1147) | 710 (361,1016) |
| **Dementia diagnosis:** % (n)* | 54.5 (152) | 56.6 (90) |
| **Depression diagnosis:** % (n)* | 56.6 (158) | 55.9 (90) |
| **Delirium diagnosis:** % (n)* | 5.7 (16) | 5.6 (9) |
| **Cognitive Skills Rating:** % (n)*^ | | |
| Severe | 28.0 (78) | 34.6 (55) |
| Moderate | 39.8 (111) | 36.5 (58) |
| Mild | 27.6 (77) | 28.9 (46) |
| None or minimal | 2.9 (8) | 0.0 (0) |
| **Activities of Daily Living care requirement:** % (n)*^ | 65.9 (184) | 72.1 (116) |
| High | 26.5 (74) | 22.4 (36) |
| Medium | 6.5 (18) | 5.6 (9) |
| Low | | |
| **Cognition and Behaviour care requirement:** % (n)*^ | 47.0 (131) | 46.0 (74) |
| High | 33.0 (92) | 38.5 (62) |
| Medium | 17.5 (49) | 15.5 (25) |
| Low | | |
| **Complex Healthcare care requirement:** % (n)*^ | | |
| High | 64.5 (180) | 67.1 (108) |
| Medium | 28.3 (79) | 29.2 (47) |
| Low | 6.1 (17) | 3.7 (6) |

Data are presented as median (IQR).
*extracted from Aged Care Funding Instrument data.
^GRACE missing data: cognitive skills rating, 1.7% (n=5); activities of daily living care requirement, 1.1% (n=3); cognition and behaviour care requirement, 2.5% (n=7); complex healthcare care requirement, 1.1% (n=3); healthcare services, 12.9% (n=36); medications, 18.3% (n=51); health conditions, 18.3% (n=51).

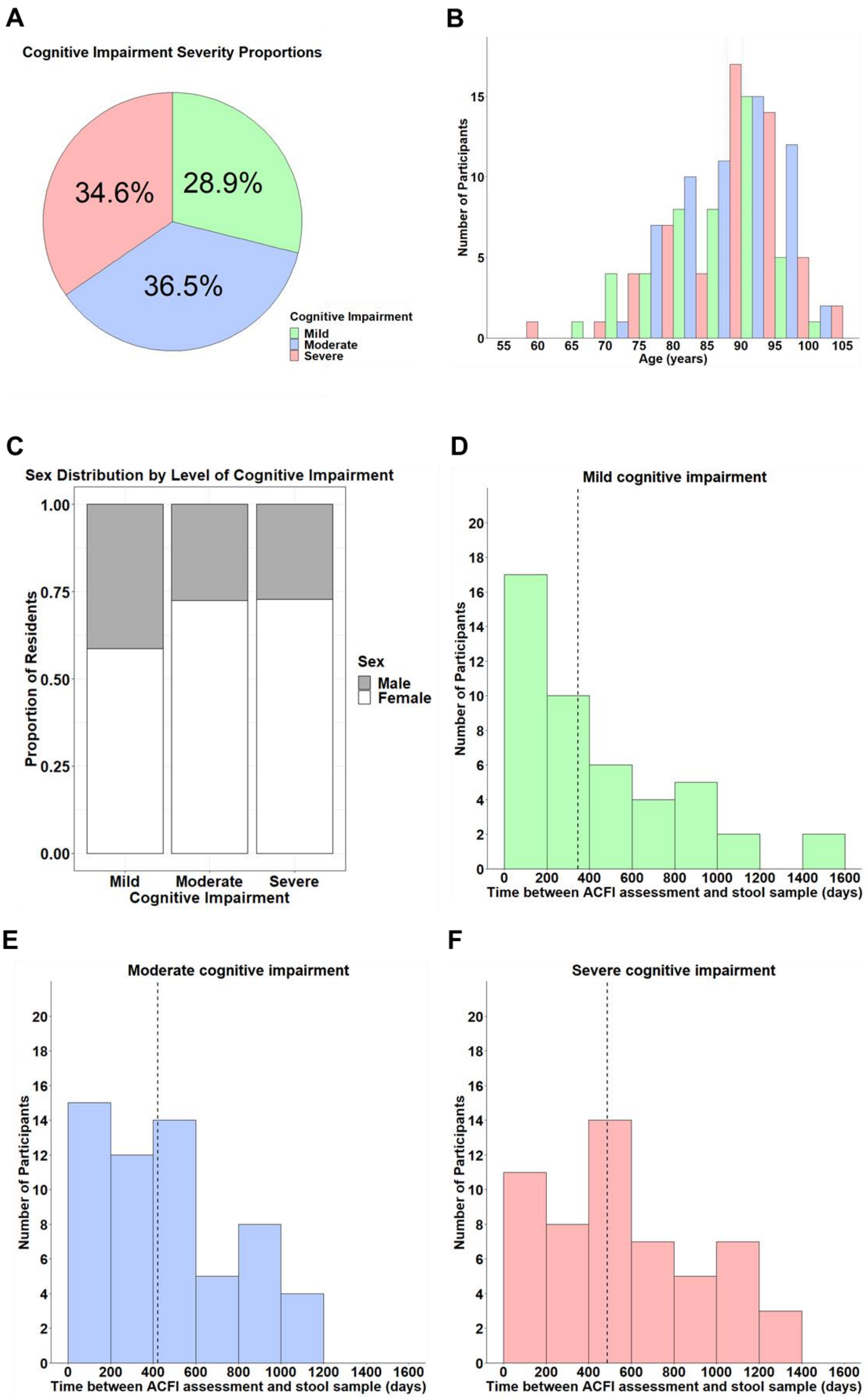


## eFigure 2. Characteristics of the cognitive impairment (CI) cohort.

A) The percentage of residents grouped by CI severity. The distribution of age (B) and sex (C) across CI groups within the CI cohort. The days between Cognitive Skills assessment and the collection of stool samples for mild (D), moderate (E), and severe (F) CI groups within the CI cohort. Mild, n=46; moderate, n=58; severe, n=55.

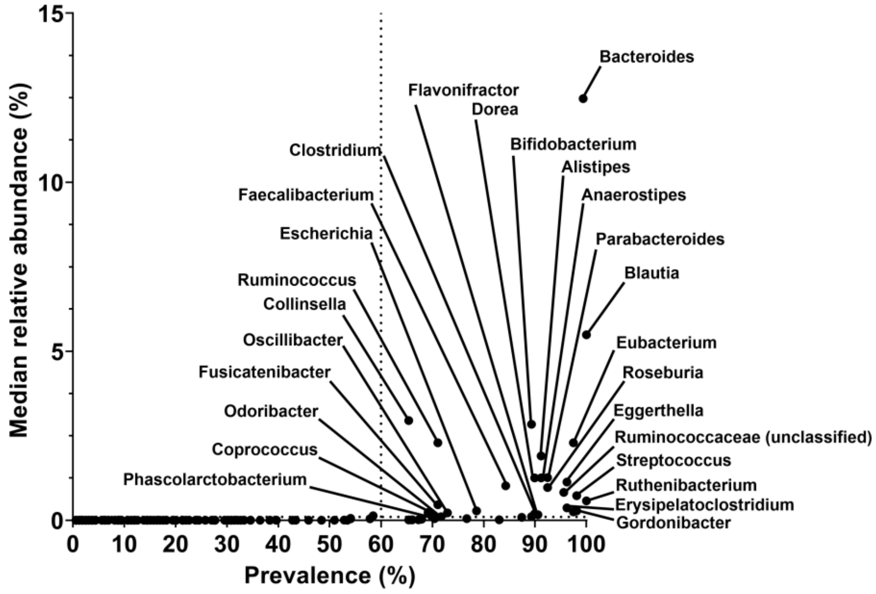


**eFigure 3. Core gut microbiome genera in residents of long-term aged care facilities.**

The frequency of genera detected and their median relative abundances, labelled with core genera.

A

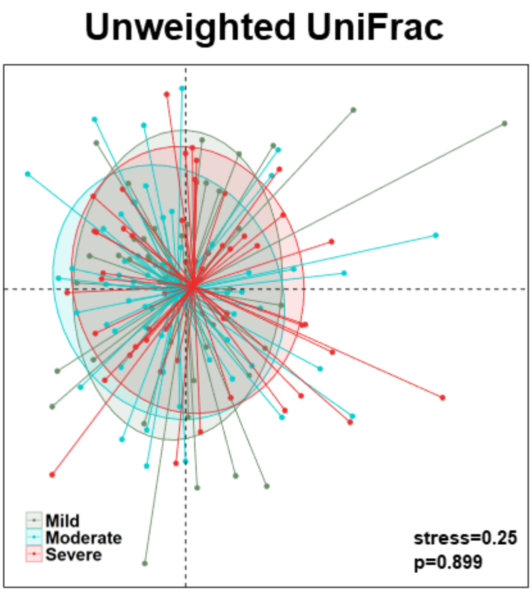


B

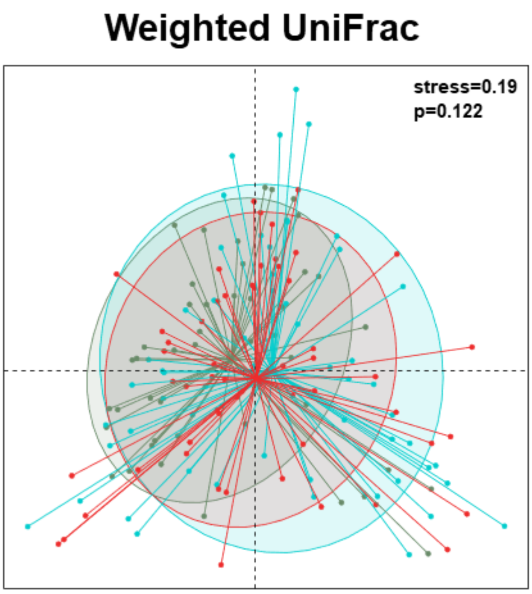


**eFigure 4. Non-metric multidimensional scaling plots of UniFrac dissimilarity of residents of long-term aged care facilities by cognitive impairment.**
**A**) Unweighted UniFrac. **B**) Weighted UniFrac. P-values determined by unadjusted PERMANOVA. Cognitive impairment categorised as mild, n=46; moderate, n=58; severe, n=55.

**eTable 2. Prevalence and relative abundance of species identified as core (present in >60% of participants) in the gut microbiome of residents of long-term aged care facilities with mild, moderate, and severe cognitive impairment (CI).**

Taxa not identified in a CI group were below detected threshold. CI; cognitive impairment.

| Species name | Mild CI | | Moderate CI | | Severe CI | |
|---|---|---|---|---|---|---|
| | Prevalence (%) | Median abundance % (range) | Prevalence (%) | Median abundance % (range) | Prevalence (%) | Median abundance % (range) |
| *Eubacterium eligens* | 60.9 | 0.18 (0, 5.2) | - | - | 73.2 | 0.13 (0, 5.5) |
| *Eubacterium rectale* | 63.0 | 0.13 (0, 19.1) | - | - | 62.5 | 0.11 (0, 18.7) |
| *Bacteroides caccae* | - | - | 61.0 | 0.23 (0, 6.4) | - | - |
| *Akkermansia muciniphila* | - | - | 62.7 | 0.18 (0, 28.4) | - | - |
| *Blautia sp CAG 257* | - | - | 64.4 | 0.16 (0, 22.2) | - | - |
| *Firmicutes bacterium CAG 83* | - | - | 64.4 | 0.16 (0, 11.3) | 78.6 | 0.18 (0, 13.5) |
| *Dorea formicigenerans* | 63.0 | 0.24 (0, 4.7) | 66.1 | 0.20 (0, 6.3) | 83.9 | 0.28 (0, 3.9) |
| *Bacteroides vulgatus* | 63.0 | 0.29 (0, 13.4) | 69.5 | 1.05 (0, 31.4) | 83.9 | 1.40 (0, 10.3) |
| *Methanobrevibacter smithii* | - | - | - | - | 64.3 | 0.62 (0, 51.4) |
| *Roseburia faecis* | 65.2 | 0.11 (0, 8.7) | 64.4 | 0.19 (0, 30.9) | - | - |
| *Bacteroides thetaiotaomicron* | 67.4 | 0.10 (0, 8.7) | 84.7 | 0.43 (0, 12.2) | 85.7 | 0.11 (0, 5.3) |
| *Fusicatenibacter saccharivorans* | 67.4 | 0.61 (0, 12.3) | 76.3 | 0.58 (0, 8.4) | 67.9 | 0.25 (0, 11.2) |
| *Bacteroides dorei* | 67.4 | 0.69 (0, 22.6) | 62.7 | 0.40 (0, 13.2) | 76.8 | 0.29 (0, 5.2) |
| *Parabacteroides merdae* | - | - | 67.8 | 0.25 (0, 10.5) | 75.0 | 0.34 (0, 8.9) |
| *Eubacterium hallii* | 69.6 | 0.11 (0, 3.1) | 69.5 | 0.13 (0, 12.5) | 89.3 | 0.22 (0, 4.7) |
| *Odoribacter splanchnicus* | 69.6 | 0.13 (0, 1.3) | - | - | - | - |
| *Alistipes putredinis* | 69.6 | 1.03 (0, 7.4) | 74.6 | 0.76 (0, 4.8) | 78.6 | 0.61 (0, 6.1) |
| *Escherichia coli* | 71.7 | 0.20 (0, 16.5) | 79.7 | 0.39 (0, 43.6) | 83.9 | 0.23 (0, 19.6) |
| *Bifidobacterium longum* | 71.7 | 1.08 (0, 35.6) | 64.4 | 0.32 (0, 34.9) | 73.2 | 1.71 (0, 60.2) |
| *Collinsella aerofaciens* | - | - | - | - | 73.2 | 4.91 (0, 47.5) |
| *Firmicutes bacterium_CAG_83* | 73.9 | 0.35 (0, 9.6) | - | - | - | - |
| *Agathobaculum butyriciproducens* | - | - | 74.6 | 0.16 (0, 6.9) | - | - |
| *Anaerostipes hadrus* | 78.3 | 1.27 (0, 14.1) | 72.9 | 1.08 (0, 17.5) | 94.6 | 1.30 (0, 34.4) |
| *Alistipes finegoldii* | - | - | 76.3 | 0.21 (0, 17.5) | 82.1 | 0.26 (0, 14.3) |
| *Erysipelatoclostridium ramosum* | - | - | 79.7 | 0.10 (0, 9.1) | - | - |

| | | | | | | |
|---|---|---|---|---|---|---|
| *Faecalibacterium prausnitzii* | 80.4 | 1.62 (0, 19.0) | 79.7 | 1.17 (0, 17.9) | 92.9 | 0.43 (0, 8.6) |
| *Blautia wexlerae* | 82.6 | 0.15 (0, 13.0) | 79.7 | 0.20 (0, 9.4) | - | - |
| *Streptococcus salivarius* | 82.6 | 0.33 (0, 7.3) | 81.4 | 0.18 (0, 12.7) | - | - |
| *Eubacterium siraeum* | 82.6 | 0.42 (0, 5.0) | 74.6 | 0.39 (0, 10.3) | 82.1 | 0.35 (0, 9.4) |
| *Blautia obeum* | 84.8 | 0.54 (0, 8.4) | 78.0 | 0.45 (0, 8.1) | 92.9 | 0.54 (0, 8.2) |
| *Parabacteroides distasonis* | 84.8 | 0.62 (0, 6.1) | 86.4 | 0.67 (0, 13.4) | 92.9 | 0.54 (0, 7.9) |
| *Ruminococcus gnavus* | 87.0 | 0.58 (0, 53.1) | 88.1 | 0.83 (0, 19.9) | 91.1 | 0.41 (0, 15.1) |
| *Streptococcus parasanguinis* | 89.1 | 0.11 (0, 4.6) | - | - | - | - |
| *Bacteroides uniformis* | 89.1 | 2.89 (0, 23.0) | 88.1 | 2.92 (0, 28.2) | 89.3 | 1.36 (0, 19.9) |
| *Clostridium leptum* | 91.3 | 0.11 (0, 1.4) | - | - | 96.4 | 0.17 (0, 1.9) |
| *Flavonifractor plautii* | 91.3 | 0.15 (0, 4.2) | 91.5 | 0.23 (0, 6.4) | 89.3 | 0.11 (0, 2.4) |
| *Clostridium innocuum* | 95.7 | 0.22 (0, 13.3) | - | - | 92.9 | 0.12 (0, 4.2) |
| *Eggerthella lenta* | 95.7 | 1.25 (0, 15.6) | 96.6 | 0.80 (0, 10.8) | 96.4 | 1.43 (0, 18.9) |
| *Gordonibacter pamelaeae* | 97.8 | 0.25 (0, 2.7) | 98.3 | 0.19 (0, 5.4) | 98.2 | 0.43 (0, 3.9) |
| *Ruthenibacterium lactatiformans* | 100.0 | 0.53 (0, 10.9) | 100.0 | 0.42 (0, 8.9) | 100.0 | 0.82 (0, 12.3) |

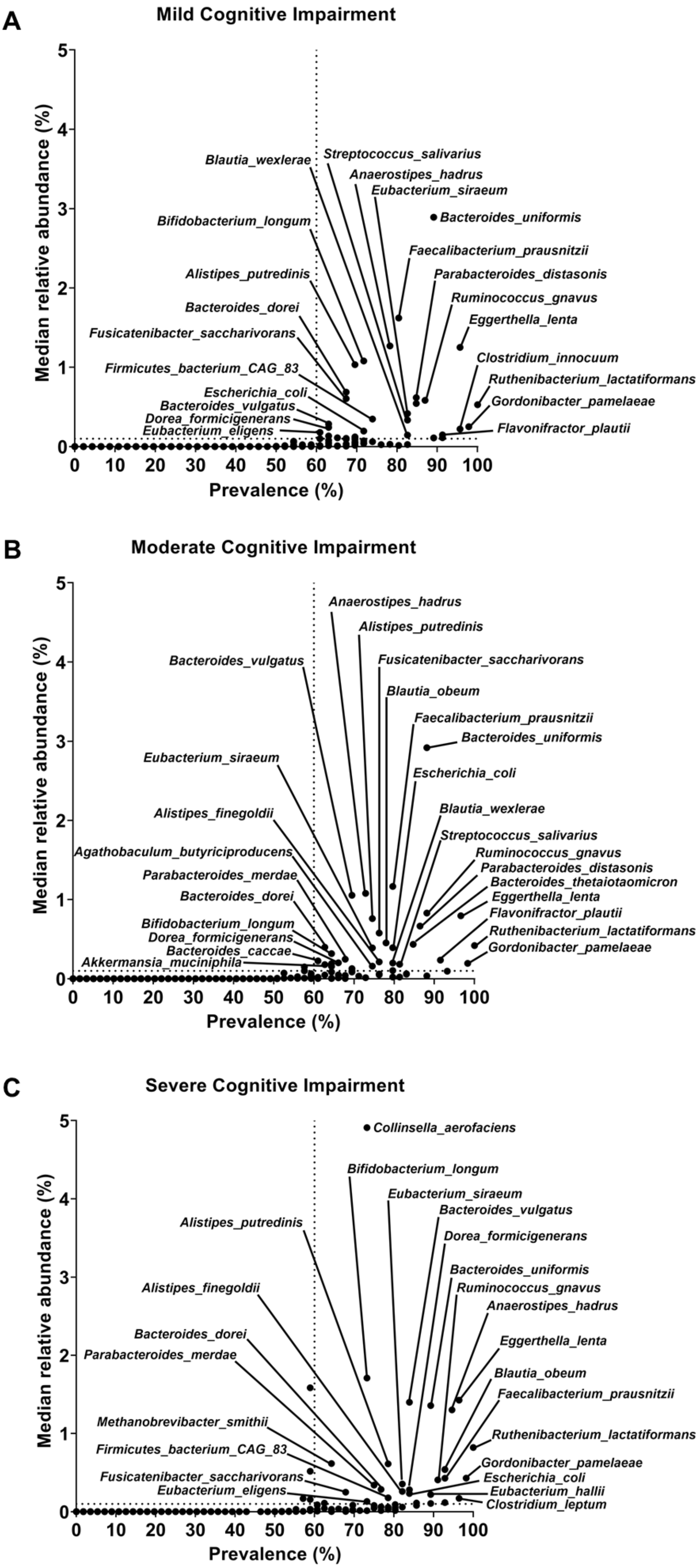
A
Mild Cognitive Impairment
Median relative abundance (%)
Prevalence (%)
Blautia_wexlerae
Streptococcus_salivarius
Anaerostipes_hadrus
Eubacterium_siraeum
Bacteroides_uniformis
Bifidobacterium_longum
Faecalibacterium_prausnitzii
Alistipes_putredinis
Parabacteroides_distasonis
Ruminococcus_gnavus
Bacteroides_dorei
Eggerthella_lenta
Fusicatenibacter_saccharivorans
Clostridium_innocuum
Firmicutes_bacterium_CAG_83
Ruthenibacterium_lactatiformans
Escherichia_coli
Gordonibacter_pamelaeae
Bacteroides_vulgatus
Dorea_formicigenerans
Flavonifractor_plautii
Eubacterium_eligens
B
Moderate Cognitive Impairment
Median relative abundance (%)
Prevalence (%)
Anaerostipes_hadrus
Alistipes_putredinis
Bacteroides_vulgatus
Fusicatenibacter_saccharivorans
Blautia_obeum
Faecalibacterium_prausnitzii
Bacteroides_uniformis
Eubacterium_siraeum
Escherichia_coli
Alistipes_finegoldii
Blautia_wexlerae
Streptococcus_salivarius
Agathobaculum_butyriciproducens
Ruminococcus_gnavus
Parabacteroides_distasonis
Parabacteroides_merdae
Bacteroides_thetaiotaomicron
Bacteroides_dorei
Eggerthella_lenta
Flavonifractor_plautii
Bifidobacterium_longum
Dorea_formicigenerans
Ruthenibacterium_lactatiformans
Bacteroides_caccae
Gordonibacter_pamelaeae
Akkermansia_muciniphila
C
Severe Cognitive Impairment
Median relative abundance (%)
Prevalence (%)
Collinsella_aerofaciens
Bifidobacterium_longum
Eubacterium_siraeum
Bacteroides_vulgatus
Alistipes_putredinis
Dorea_formicigenerans
Bacteroides_uniformis
Alistipes_finegoldii
Ruminococcus_gnavus
Anaerostipes_hadrus
Bacteroides_dorei
Eggerthella_lenta
Parabacteroides_merdae
Blautia_obeum
Faecalibacterium_prausnitzii
Methanobrevibacter_smithii
Ruthenibacterium_lactatiformans
Firmicutes_bacterium_CAG_83
Gordonibacter_pamelaeae
Fusicatenibacter_saccharivorans
Escherichia_coli
Eubacterium_eligens
Eubacterium_hallii
Clostridium_leptum

## eFigure 5. Core species profiles by cognitive impairment (CI) severity.

The frequency of species detected in the gut microbiome and their median relative abundances for: **A**) mild CI, **B**) moderate CI, and **C**) severe CI. Labelled species above core threshold.

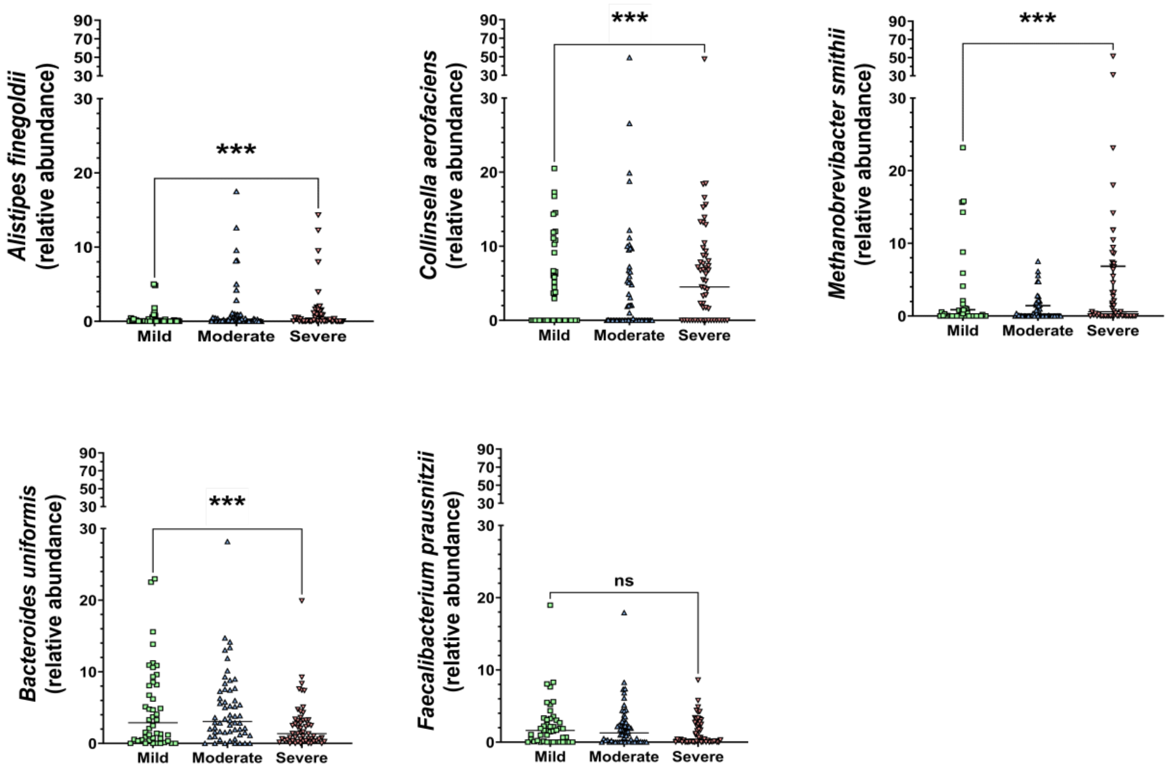


## eFigure 6. Species-level differences in the gut microbiome of residents of long-term aged care by cognitive impairment.

The relative abundance of bacterial species associated with cognitive impairment severity and aging: *Alistipes finegoldii*, *Collinsella aerofaciens*, *Methanobrevibacter smithii*, *Bacteroides uniformis*, and *Faecalibacterium prausnitzii*. Performed by multivariate analysis treating CI severity as a categorical variable and adjusting for time since cognitive impairment assessment, age, sex, antibiotic use, proton pump inhibitor use, opioid use, laxative use, recorded medical history, meal texture, and liquid texture. Categorical plots illustrate significance of this multivariate analysis between mild and severe cognitive impairment, where mild was the reference group. ns=not significant; *q<0.05; **q<0.01; ***q<0.001 for adjusted p-values following FDR correction. Mild, n=46; moderate, n=58; severe, n=55.

**eTable 3. The differences in abundance of microbial functional capacity for all mapped metagenomic pathways at the whole community level in residents of long-term aged care facilities with increasingly severe CI, at statistical significance.**

The magnitude of the difference is presented as highest to lowest odds ratio with 95% confidence intervals following a multivariate adjustment for time since cognitive impairment assessment, age, sex, antibiotic use, proton pump inhibitor use, opioid use, laxative use, recorded medical history, meal texture, and liquid texture. Pathways of interest identified in bold.

| Odds ratio | Conf. int. low | Conf. int. high | Pathway | P-value | FDR |
|---|---|---|---|---|---|
| 1.113094 | 1.078745838 | 1.149154726 | PWY.7286..7..3.amino.3.carboxypropyl..wyosine.biosynthesis | 2.99E-11 | 6.33E-10 |
| 1.107933 | 1.073984937 | 1.143529212 | PWY.8112..factor.420.biosynthesis.I..archaea. | 1.48E-10 | 2.20E-09 |
| 1.107342 | 1.073282852 | 1.143069752 | PWY.6350..archaetidylinositol.biosynthesis | 2.17E-10 | 3.11E-09 |
| 1.105943 | 1.071803128 | 1.141740534 | PWY.8113..3PG.factor.420.biosynthesis | 4.14E-10 | 5.04E-09 |
| 1.104314 | 1.070596543 | 1.139645541 | PWY.5198..factor.420.biosynthesis.II..mycobacteria. | 4.75E-10 | 5.62E-09 |
| 1.10393 | 1.068642914 | 1.141098788 | PWY.6167..flavin.biosynthesis.II..archaea. | 3.37E-09 | 2.60E-08 |
| 1.099599 | 1.06632844 | 1.134415866 | PWY.6349..CDP.archaeol.biosynthesis | 1.78E-09 | 1.60E-08 |
| 1.097014 | 1.064550378 | 1.130712389 | PWY.7198..pyrimidine.deoxyribonucleotides.de.novo.biosynthesis.IV | 1.61E-09 | 1.58E-08 |
| 1.096986 | 1.063753135 | 1.131746296 | PWY.6160..3.dehydroquinate.biosynthesis.II..archaea. | 4.62E-09 | 3.44E-08 |
| 1.096986 | 1.063753135 | 1.131746296 | PWY.6165..chorismate.biosynthesis.II..archaea. | 4.62E-09 | 3.44E-08 |
| 1.091733 | 1.058434844 | 1.126579998 | METHANOGENESIS.PWY..methanogenesis.from.H2.and.CO2 | 3.44E-08 | 2.13E-07 |
| 1.08915 | 1.055992566 | 1.123841604 | PWY.5209..methyl.coenzyme.M.oxidation.to.CO2 | 7.48E-08 | 3.80E-07 |
| 1.088497 | 1.055950561 | 1.122428967 | PWY.8187..L.arginine.degradation.XIII..reductive.Stickland.reaction. | 4.73E-08 | 2.72E-07 |
| 1.087369 | 1.051848025 | 1.124795631 | PWY.7159..3.8.divinyl.chlorophyllide.a.biosynthesis.III..aerobic..light.independent. | 9.56E-07 | 3.77E-06 |
| 1.084402 | 1.048690756 | 1.122071647 | PWY.7031..protein.N.glycosylation..bacterial. | 2.60E-06 | 9.24E-06 |
| 1.071246 | 1.037109738 | 1.107020183 | PWY.6518..bile.acids.epimerization | 3.49E-05 | 9.81E-05 |

| | | | | | |
|---|---|---|---|---|---|
| 1.063492 | 1.033363444 | 1.094611743 | PWY.7383..anaerobic.energy.metabolism..invertebrates..cytosol. | 2.80E-05 | 7.98E-05 |
| 1.059425 | 1.026396989 | 1.093948956 | PWY.5531..3.8.divinyl.chlorophyllide.a.biosynthesis.II..anaerobic. | 0.000381 | 0.000816 |
| 1.055535 | 1.024389794 | 1.087561131 | UDPNAGSYN.PWY..UDP.N.acetyl.D.glucosamine.biosynthesis.I | 0.000358 | 0.000778 |
| **1.048846** | **1.018965188** | **1.079828715** | **HISTSYN.PWY..L.histidine.biosynthesis** | **0.001238** | **0.002439** |
| 1.047155 | 1.017568965 | 1.077756687 | PWY.7209..superpathway.of.pyrimidine.ribonucleosides.degradation | 0.001662 | 0.003151 |
| **1.047085** | **1.016801803** | **1.078582718** | **PWY.2942..L.lysine.biosynthesis.III** | **0.002254** | **0.004118** |
| 1.046363 | 1.015822864 | 1.077824369 | PWY.1861..formaldehyde.assimilation.II..assimilatory.RuMP.Cycle. | 0.002573 | 0.004638 |
| 1.046028 | 1.016334804 | 1.07668889 | PWY66.399..gluconeogenesis.III | 0.002199 | 0.004037 |
| 1.043559 | 1.01408131 | 1.074014708 | RUMP.PWY..formaldehyde.oxidation.I | 0.003588 | 0.006298 |
| **1.043065** | **1.013395656** | **1.073679943** | **P124.PWY..Bifidobacterium.shunt** | **0.004213** | **0.007301** |
| 1.038445 | 1.008157506 | 1.069885067 | NONMEVIPP.PWY..methylerythritol.phosphate.pathway.I | 0.01286 | 0.020763 |
| 1.038307 | 1.008513155 | 1.069171527 | OANTIGEN.PWY..O.antigen.building.blocks.biosynthesis..E..coli. | 0.011886 | 0.019345 |
| 1.036371 | 1.007197838 | 1.066550144 | FASYN.INITIAL.PWY..superpathway.of.fatty.acid.biosynthesis.initiation..E..coli. | 0.014391 | 0.022866 |
| 1.033883 | 1.003744129 | 1.064470963 | PWY.5686..UMP.biosynthesis.I | 0.02524 | 0.038145 |
| 1.033883 | 1.003744129 | 1.064470963 | PWY.7790..UMP.biosynthesis.II | 0.02524 | 0.038145 |
| 1.033883 | 1.003744129 | 1.064470963 | PWY.7791..UMP.biosynthesis.III | 0.02524 | 0.038145 |
| 1.033566 | 1.004604078 | 1.063459744 | PWY.622..starch.biosynthesis | 0.022938 | 0.035466 |
| 1.032343 | 1.00311404 | 1.062512074 | PWY.6122..5.aminoimidazole.ribonucleotide.biosynthesis.II | 0.030636 | 0.045614 |
| 1.032343 | 1.00311404 | 1.062512074 | PWY.6277..superpathway.of.5.aminoimidazole.ribonucleotide.biosynthesis | 0.030636 | 0.045614 |

| | | | | | |
|---|---|---|---|---|---|
| 1.03142 | 1.001035 129 | 1.062426 454 | PWY0.162..superpathway.of.pyrimidine.ribonucleotides.de.novo.biosynthesis | 0.0408 79 | 0.0599 76 |
| 1.0289 | 1.000346 374 | 1.058372 68 | PWY.7371..1.4.dihydroxy.6.naphthoate.biosynthesis.II | 0.0475 11 | 0.0694 52 |
| 0.969588 | 0.941716 645 | 0.998138 128 | BIOTIN.BIOSYNTHESIS.PWY..biotin.biosynthesis.I | 0.0373 5 | 0.0549 99 |
| 0.969585 | 0.941921 933 | 0.997876 905 | PWY.7688..dTDP..alpha..D.ravidosamine.and.dTDP.4.acetyl..alpha..D.ravidosamine.biosynthesi s | 0.0358 17 | 0.0529 35 |
| 0.969214 | 0.941398 871 | 0.997459 178 | ILEUSYN.PWY..L.isoleucine.biosynthesis.I..from.threonine. | 0.0325 65 | 0.0483 07 |
| 0.968041 | 0.940604 831 | 0.996227 949 | PWY0.42..2.methylcitrate.cycle.I | 0.0266 11 | 0.0400 67 |
| **0.967749** | **0.940363 321** | **0.995628 835** | **VALSYN.PWY..L.valine.biosynthesis** | **0.0247 57** | **0.0379 49** |
| 0.967703 | 0.939545 278 | 0.996559 304 | PWY.2941..L.lysine.biosynthesis.II | 0.0288 71 | 0.0433 06 |
| 0.967504 | 0.939900 562 | 0.995767 549 | PWY0.1479..tRNA.processing | 0.0248 27 | 0.0379 49 |
| 0.967242 | 0.939641 574 | 0.995662 934 | PWY.6124..inosine.5..phosphate.biosynthesis.II | 0.0242 52 | 0.0373 53 |
| 0.966689 | 0.940295 831 | 0.993704 318 | TCA..TCA.cycle.I..prokaryotic. | 0.0161 69 | 0.0253 91 |
| 0.966435 | 0.938374 937 | 0.995156 655 | PYRIDNUCSYN.PWY..NAD.de.novo.biosynthesis.I..from.aspartate. | 0.0223 29 | 0.0346 57 |
| 0.966329 | 0.938587 333 | 0.994730 975 | PWY.5913..partial.TCA.cycle..obligate.autotrophs. | 0.0209 15 | 0.0325 89 |
| 0.964887 | 0.937034 347 | 0.993375 047 | PWY.6317..D.galactose.degradation.I..Leloir.pathway. | 0.0163 53 | 0.0255 8 |
| 0.964486 | 0.937732 766 | 0.991929 347 | PWY.5464..superpathway.of.cytosolic.glycolysis..plants...pyruvate.dehydrogenase.and.TCA.cycl e | 0.0115 98 | 0.0189 52 |
| 0.964435 | 0.936737 231 | 0.992762 791 | PWY.7560..methylerythritol.phosphate.pathway.II | 0.0143 3 | 0.0228 6 |
| 0.964054 | 0.935852 852 | 0.993048 67 | PWY0.461..L.lysine.degradation.I | 0.0154 92 | 0.0244 23 |
| 0.963519 | 0.935056 575 | 0.992411 331 | METH.ACETATE.PWY..methanogenesis.from.acetate | 0.0144 51 | 0.0228 71 |
| 0.963426 | 0.935512 496 | 0.991780 565 | PWY.5030..L.histidine.degradation.III | 0.0119 73 | 0.0194 08 |

| | | | | | |
|---|---|---|---|---|---|
| **0.962978** | **0.934421976** | **0.992233111** | **PWY.7400..L.arginine.biosynthesis.IV..archaebacteria.** | **0.013948** | **0.022339** |
| 0.962568 | 0.934827391 | 0.99087768 | GLYCOLYSIS.E.D..superpathway.of.glycolysis.and.the.Entner.Doudoroff.pathway | 0.010325 | 0.017011 |
| 0.962343 | 0.935705373 | 0.989597444 | PWY.5971..palmitate.biosynthesis..type.II.fatty.acid.synthase. | 0.007181 | 0.012128 |
| 0.962311 | 0.934723876 | 0.99044593 | PWY.8004..Entner.Doudoroff.pathway.I | 0.00936 | 0.015485 |
| 0.962182 | 0.933939058 | 0.991052114 | PWY.8131..5..deoxyadenosine.degradation.II | 0.010933 | 0.017938 |
| 0.962017 | 0.934376285 | 0.989983304 | PWY.5121..superpathway.of.geranylgeranyl.diphosphate.biosynthesis.II..via.MEP. | 0.009178 | 0.015246 |
| 0.961895 | 0.934741686 | 0.989673275 | METHGLYUT.PWY..superpathway.of.methylglyoxal.degradation | 0.007622 | 0.012768 |
| 0.961826 | 0.932533388 | 0.991952856 | PWY0.1277..3.phenylpropanoate.and.3..3.hydroxyphenyl.propanoate.degradation | 0.01344 | 0.021612 |
| **0.961622** | **0.933939652** | **0.990023986** | **GLUDEG.I.PWY..GABA.shunt** | **0.00849** | **0.014161** |
| 0.959655 | 0.932656756 | 0.987205741 | PWY.7210..pyrimidine.deoxyribonucleotides.biosynthesis.from.CTP | 0.00449 | 0.007649 |
| 0.958615 | 0.931140518 | 0.986706767 | GALACT.GLUCUROCAT.PWY..superpathway.of.hexuronide.and.hexuronate.degradation | 0.004236 | 0.007308 |
| **0.957995** | **0.930515044** | **0.985872943** | **HSERMETANA.PWY..L.methionine.biosynthesis.III** | **0.00375** | **0.006554** |
| 0.957931 | 0.930965176 | 0.985552988 | PWY.5705..allantoin.degradation.to.glyoxylate.III | 0.003091 | 0.005547 |
| 0.95781 | 0.930459441 | 0.985713768 | PWY.6588..pyruvate.fermentation.to.acetone | 0.00345 | 0.006136 |
| 0.957731 | 0.930293739 | 0.985933856 | PENTOSE.P.PWY..pentose.phosphate.pathway | 0.003515 | 0.006197 |
| 0.957721 | 0.927748097 | 0.988465074 | PWY0.41..allantoin.degradation.IV..anaerobic. | 0.007513 | 0.012638 |
| 0.957326 | 0.929264544 | 0.986125848 | PWY.5747..2.methylcitrate.cycle.II | 0.003976 | 0.00692 |
| 0.957121 | 0.928527455 | 0.985839714 | PWY.7456...beta...1.4..mannan.degradation | 0.003465 | 0.006136 |
| 0.957094 | 0.930216323 | 0.984632712 | PWY.5265..peptidoglycan.biosynthesis.II..staphylococci. | 0.002484 | 0.004499 |

| | | | | | |
|---|---|---|---|---|---|
| **0.956933** | **0.927582651** | **0.986969482** | **PWY.8190..L.glutamate.degradation.XI..reductive.Stickland.reaction.** | **0.005381** | **0.009127** |
| 0.956298 | 0.92959361 | 0.983608423 | PWY.6803..phosphatidylcholine.acyl.editing | 0.001916 | 0.003599 |
| 0.956264 | 0.927260814 | 0.986064367 | HCAMHPDEG.PWY..3.phenylpropanoate.and.3..3.hydroxyphenyl.propanoate.degradation.to.2.hydroxypentadienoate | 0.004326 | 0.007401 |
| 0.956264 | 0.927260814 | 0.986064367 | PWY.6690..cinnamate.and.3.hydroxycinnamate.degradation.to.2.hydroxypentadienoate | 0.004326 | 0.007401 |
| 0.956034 | 0.929808582 | 0.982871824 | TCA.GLYOX.BYPASS..superpathway.of.glyoxylate.bypass.and.TCA | 0.001487 | 0.002859 |
| 0.95588 | 0.928815746 | 0.983477673 | GALACTUROCAT.PWY..D.galacturonate.degradation.I | 0.002027 | 0.003773 |
| 0.95581 | 0.929463589 | 0.982754829 | GLYOXYLATE.BYPASS..glyoxylate.cycle | 0.001476 | 0.002853 |
| 0.955793 | 0.92819955 | 0.98387475 | PWY.6700..queuosine.biosynthesis.I..de.novo. | 0.002268 | 0.004125 |
| 0.955432 | 0.926733691 | 0.984739237 | PWY.7388..octanoyl..acyl.carrier.protein..biosynthesis..mitochondria..yeast. | 0.003219 | 0.005752 |
| 0.955409 | 0.928613424 | 0.982856921 | P221.PWY..octane.oxidation | 0.001626 | 0.003097 |
| 0.954984 | 0.927369188 | 0.98334059 | SALVADEHYPOX.PWY..adenosine.nucleotides.degradation.II | 0.002061 | 0.003818 |
| **0.954964** | **0.927383303** | **0.983157483** | **PWY.6292..superpathway.of.L.cysteine.biosynthesis..mammalian.** | **0.001969** | **0.003682** |
| 0.954719 | 0.927154018 | 0.982916107 | PWY1ZNC.1..assimilatory.sulfate.reduction.IV | 0.001858 | 0.003507 |
| **0.954621** | **0.927200818** | **0.9825786** | **CITRULBIO.PWY..L.citrulline.biosynthesis** | **0.001572** | **0.003009** |
| 0.954565 | 0.926570132 | 0.983092479 | PWY.7761..NAD.salvage.pathway.II..PNC.IV.cycle. | 0.002131 | 0.00393 |
| 0.953867 | 0.926536312 | 0.981798419 | PWY0.1241..ADP.L.glycero..beta..D.manno.heptose.biosynthesis | 0.001385 | 0.002704 |
| 0.953832 | 0.926361388 | 0.981961134 | PWY.7242..D.fructuronate.degradation | 0.001452 | 0.002819 |
| 0.953767 | 0.926520056 | 0.981634967 | REDCITCYC..TCA.cycle.VI..Helicobacter. | 0.001314 | 0.002577 |
| 0.952009 | 0.92466549 | 0.979954988 | PWY.6168..flavin.biosynthesis.III..fungi. | 0.000895 | 0.0018 |

| | | | | | |
|---|---|---|---|---|---|
| 0.951625 | 0.923655525 | 0.980163104 | PWY.6527..stachyose.degradation | 0.001084 | 0.002147 |
| 0.951438 | 0.923608105 | 0.980006655 | PWY.7237..myo...chiro..and.scyllo.inositol.degradation | 0.000988 | 0.001966 |
| 0.950764 | 0.923994321 | 0.978166781 | PWY.5675..nitrate.reduction.V..assimilatory. | 0.00051 | 0.001062 |
| 0.95047 | 0.92269037 | 0.978907233 | PWY4LZ.257..superpathway.of.fermentation..Chlamydomonas.reinhardtii. | 0.000755 | 0.001532 |
| **0.950333** | **0.922225424** | **0.97868403** | **PWY.702..L.methionine.biosynthesis.II** | **0.000712** | **0.001452** |
| 0.950286 | 0.923214861 | 0.9778794 | GLUCUROCAT.PWY..superpathway.of..beta..D.glucuronosides.degradation | 0.000516 | 0.00107 |
| 0.950253 | 0.924041188 | 0.977054175 | PWY.561..superpathway.of.glyoxylate.cycle.and.fatty.acid.degradation | 0.000334 | 0.000734 |
| 0.950074 | 0.922039912 | 0.978769243 | SULFATE.CYS.PWY..superpathway.of.sulfate.assimilation.and.cysteine.biosynthesis | 0.000767 | 0.00155 |
| 0.950041 | 0.922673568 | 0.978056242 | P125.PWY..superpathway.of..R.R..butanediol.biosynthesis | 0.000564 | 0.001163 |
| 0.94947 | 0.922343531 | 0.977169419 | PWY.241..C4.photosynthetic.carbon.assimilation.cycle..NADP.ME.type | 0.000449 | 0.000939 |
| 0.949432 | 0.922516972 | 0.976948167 | PWY.5918..superpathway.of.heme.b.biosynthesis.from.glutamate | 0.000385 | 0.000816 |
| 0.949173 | 0.922190939 | 0.976786006 | P23.PWY..reductive.TCA.cycle.I | 0.000375 | 0.000811 |
| 0.948887 | 0.922108087 | 0.976240472 | PWY.7184..pyrimidine.deoxyribonucleotides.de.novo.biosynthesis.I | 0.00031 | 0.000688 |
| 0.948884 | 0.92181819 | 0.976562705 | PWY.4041...gamma..glutamyl.cycle | 0.000357 | 0.000778 |
| 0.948686 | 0.921374227 | 0.976596912 | PWY.7315..dTDP.N.acetylthomosamine.biosynthesis | 0.000386 | 0.000816 |
| **0.948678** | **0.922207516** | **0.97573389** | **PWY.6922..L.N.delta..acetylornithine.biosynthesis** | **0.00025** | **0.000564** |
| **0.947645** | **0.919701081** | **0.976237995** | **PWY.5345..superpathway.of.L.methionine.biosynthesis..by.sulfhydrylation.** | **0.000407** | **0.000857** |
| 0.94753 | 0.919640745 | 0.976064915 | SO4ASSIM.PWY..assimilatory.sulfate.reduction.I | 0.000385 | 0.000816 |
| 0.947489 | 0.919784469 | 0.975335829 | CALVIN.PWY..Calvin.Benson.Bassham.cycle | 0.000271 | 0.000608 |

| | | | | | |
|---|---|---|---|---|---|
| 0.947377 | 0.920347257 | 0.974971456 | PWY.5189..tetrapyrrole.biosynthesis.II..from.glycine. | 0.000236 | 0.000535 |
| 0.947289 | 0.921077445 | 0.974093064 | AST.PWY..L.arginine.degradation.II..AST.pathway. | 0.000148 | 0.000353 |
| 0.946934 | 0.918685014 | 0.975342264 | PWY.6606..guanosine.nucleotides.degradation.II | 0.000316 | 0.000699 |
| 0.946646 | 0.91700856 | 0.976876176 | PWY66.430..myristate.biosynthesis..mitochondria. | 0.00067 | 0.001374 |
| 0.94662 | 0.919974632 | 0.973875998 | PWY.5723..Rubisco.shunt | 0.000158 | 0.000369 |
| 0.946275 | 0.918640919 | 0.974078803 | THISYNARA.PWY..superpathway.of.thiamine.diphosphate.biosynthesis.III..eukaryotes. | 0.000202 | 0.000463 |
| 0.946088 | 0.915348033 | 0.97761391 | PWY.7434..terminal.O.glycans.residues.modification..via.type.2.precursor.disaccharide. | 0.000953 | 0.001905 |
| 0.946055 | 0.919390128 | 0.973319831 | PWY.6284..superpathway.of.unsaturated.fatty.acids.biosynthesis..E..coli. | 0.000136 | 0.000333 |
| 0.945802 | 0.919391981 | 0.972759819 | PWY.5850..superpathway.of.menaquinol.6.biosynthesis | 0.000107 | 0.00027 |
| 0.945802 | 0.919391981 | 0.972759819 | PWY.5896..superpathway.of.menaquinol.10.biosynthesis | 0.000107 | 0.00027 |
| 0.945729 | 0.91803284 | 0.974058179 | GLYCOL.GLYOXDEG.PWY..superpathway.of.glycol.metabolism.and.degradation | 0.00022 | 0.000503 |
| 0.945726 | 0.918825408 | 0.973211502 | PWY.6906..chitin.derivatives.degradation | 0.000141 | 0.000344 |
| 0.945655 | 0.918565167 | 0.973346404 | PWY.6902..chitin.degradation.II..Vibrio. | 0.000161 | 0.000375 |
| 0.945328 | 0.91848974 | 0.972732472 | PWY.6545..pyrimidine.deoxyribonucleotides.de.novo.biosynthesis.III | 0.000122 | 0.000303 |
| 0.945248 | 0.916787691 | 0.97442452 | THREOCAT.PWY..superpathway.of.L.threonine.metabolism | 0.000292 | 0.000652 |
| 0.945215 | 0.917860168 | 0.97314409 | PWY.5497..purine.nucleobases.degradation.II..anaerobic. | 0.000158 | 0.000369 |
| 0.94517 | 0.917962312 | 0.97285609 | PWY.1042..glycolysis.IV | 0.000144 | 0.000346 |
| **0.94516** | **0.917569927** | **0.973116246** | **PWY.I9..L.cysteine.biosynthesis.VI..from.L.methionine.** | **0.000173** | **0.0004** |
| 0.944253 | 0.91663812 | 0.972427842 | PWY.6353..purine.nucleotides.degradation.II..aerobic. | 0.00015 | 0.000357 |

| | | | | | |
|---|---|---|---|---|---|
| **0.944025** | **0.916441237** | **0.972228963** | **P161.PWY..acetylene.degradation..anaerobic.** | **0.000132** | **0.000325** |
| 0.943997 | 0.915832558 | 0.972469539 | P185.PWY..formaldehyde.assimilation.III..dihydroxyacetone.cycle. | 0.000152 | 0.00036 |
| 0.943912 | 0.916328386 | 0.972110332 | PWY.7392..taxadiene.biosynthesis..engineered. | 0.000128 | 0.000316 |
| **0.943251** | **0.916620812** | **0.970478283** | **PWY.821..superpathway.of.sulfur.amino.acid.biosynthesis..Saccharomyces.cerevisiae.** | **5.99E-05** | **0.000156** |
| 0.94321 | 0.915971377 | 0.97097145 | PWY.5973..cis.vaccenate.biosynthesis | 8.73E-05 | 0.000224 |
| 0.942931 | 0.91655869 | 0.969842849 | PWY.5860..superpathway.of.demethylmenaquinol.6.biosynthesis.I | 4.53E-05 | 0.000123 |
| 0.942711 | 0.916361056 | 0.969630914 | PWY.6285..superpathway.of.fatty.acids.biosynthesis..E..coli. | 4.23E-05 | 0.000116 |
| 0.942648 | 0.915941397 | 0.969966317 | PWY.5138..fatty.acid..beta..oxidation.IV..unsaturated..even.number. | 5.30E-05 | 0.000141 |
| 0.942453 | 0.913985891 | 0.971584148 | PWY.7616..methanol.oxidation.to.carbon.dioxide | 0.000142 | 0.000344 |
| 0.942079 | 0.915283109 | 0.969505821 | FAO.PWY..fatty.acid..beta..oxidation.I..generic. | 4.79E-05 | 0.000129 |
| 0.941775 | 0.914588551 | 0.969574739 | PWY.5392..reductive.TCA.cycle.II | 5.57E-05 | 0.000147 |
| 0.94175 | 0.914256648 | 0.969754004 | POLYAMSYN.PWY..superpathway.of.polyamine.biosynthesis.I | 6.70E-05 | 0.000174 |
| 0.941357 | 0.913595347 | 0.969663734 | PWY.5384..sucrose.degradation.IV..sucrose.phosphorylase. | 7.04E-05 | 0.000181 |
| **0.940805** | **0.912435385** | **0.96975918** | **PWY.5676..acetyl.CoA.fermentation.to.butanoate.II** | **8.79E-05** | **0.000224** |
| 0.940313 | 0.912331392 | 0.968877235 | GOLPDLCAT.PWY..superpathway.of.glycerol.degradation.to.1.3.propanediol | 5.73E-05 | 0.00015 |
| 0.940221 | 0.912729308 | 0.968332509 | PWY.7345..superpathway.of.anaerobic.sucrose.degradation | 4.37E-05 | 0.000119 |
| **0.939994** | **0.912178778** | **0.968197666** | **PWY.7977..L.methionine.biosynthesis.IV** | **4.81E-05** | **0.000129** |
| 0.93994 | 0.912910862 | 0.967561407 | GLUCONEO.PWY..gluconeogenesis.I | 3.12E-05 | 8.84E-05 |
| 0.939703 | 0.912090683 | 0.967993604 | PWY.5855..ubiquinol.7.biosynthesis..early.decarboxylation. | 4.12E-05 | 0.000114 |

| | | | | | |
|---|---|---|---|---|---|
| 0.939012 | 0.912599114 | 0.965949519 | ORNDEG.PWY..superpathway.of.ornithine.degradation | 1.40E-05 | 4.17E-05 |
| **0.938887** | **0.912532986** | **0.965770297** | **P108.PWY..pyruvate.fermentation.to.propanoate.I** | **1.29E-05** | **3.90E-05** |
| 0.938824 | 0.912314936 | 0.965888267 | PWY0.1338..polymyxin.resistance | 1.43E-05 | 4.23E-05 |
| 0.938816 | 0.91071736 | 0.967171846 | PWY.6609..adenine.and.adenosine.salvage.III | 3.53E-05 | 9.85E-05 |
| 0.938433 | 0.911753531 | 0.965715722 | PWY.7118..chitin.deacetylation | 1.46E-05 | 4.29E-05 |
| 0.938359 | 0.91159511 | 0.965989399 | PWY.6895..superpathway.of.thiamine.diphosphate.biosynthesis.II | 1.67E-05 | 4.83E-05 |
| 0.93718 | 0.909735798 | 0.965138972 | PWY66.367..ketogenesis | 1.67E-05 | 4.83E-05 |
| 0.937168 | 0.910327546 | 0.964478632 | PWY.5695..inosine.5..phosphate.degradation | 1.04E-05 | 3.20E-05 |
| 0.936919 | 0.909371994 | 0.964962731 | PWY.7111..pyruvate.fermentation.to.isobutanol..engineered. | 1.75E-05 | 5.03E-05 |
| **0.936453** | **0.909204334** | **0.963825522** | **ASPASN.PWY..superpathway.of.L.aspartate.and.L.asparagine.biosynthesis** | **9.08E-06** | **2.85E-05** |
| 0.936426 | 0.909691637 | 0.963662948 | DENOVOPURINE2.PWY..superpathway.of.purine.nucleotides.de.novo.biosynthesis.II | 7.82E-06 | 2.56E-05 |
| 0.936039 | 0.908893847 | 0.963824358 | PWY.6897..thiamine.diphosphate.salvage.II | 9.97E-06 | 3.08E-05 |
| 0.93552 | 0.907935756 | 0.963610846 | PWY.6147..6.hydroxymethyl.dihydropterin.diphosphate.biosynthesis.I | 9.95E-06 | 3.08E-05 |
| 0.9355 | 0.90833836 | 0.963169708 | NAGLIPASYN.PWY..lipid.IVA.biosynthesis..E..coli. | 8.13E-06 | 2.61E-05 |
| 0.9355 | 0.90833836 | 0.963169708 | PWY.8073..lipid.IVA.biosynthesis..P..putida. | 8.13E-06 | 2.61E-05 |
| 0.935346 | 0.907465359 | 0.963765996 | PWY0.1296..purine.ribonucleosides.degradation | 1.37E-05 | 4.11E-05 |
| 0.935273 | 0.907654649 | 0.963370474 | PWY.6731..starch.degradation.III | 1.09E-05 | 3.31E-05 |
| 0.935033 | 0.908084077 | 0.962568914 | PWY.5367..petroselinate.biosynthesis | 6.15E-06 | 2.05E-05 |
| **0.934567** | **0.90767576** | **0.962031568** | **PWY.5022..4.aminobutanoate.degradation.V** | **5.03E-06** | **1.73E-05** |

| | | | | | |
|---|---|---|---|---|---|
| 0.934491 | 0.907999631 | 0.961561623 | PWY0.1337..oleate..beta..oxidation | 3.54E-06 | 1.24E-05 |
| 0.934257 | 0.906596754 | 0.962398837 | ARG.POLYAMINE.SYN..superpathway.of.arginine.and.polyamine.biosynthesis | 6.96E-06 | 2.29E-05 |
| 0.934144 | 0.906846541 | 0.96198656 | PWY.6703..preQ0.biosynthesis | 6.11E-06 | 2.05E-05 |
| 0.933788 | 0.907511929 | 0.96062417 | PWY.7942..5.oxo.L.proline.metabolism | 2.32E-06 | 8.31E-06 |
| 0.933711 | 0.90583812 | 0.962244701 | PWY0.1319..CDP.diacylglycerol.biosynthesis.II | 8.29E-06 | 2.62E-05 |
| 0.933711 | 0.90583812 | 0.962244701 | PWY.5667..CDP.diacylglycerol.biosynthesis.I | 8.29E-06 | 2.62E-05 |
| 0.933506 | 0.906855271 | 0.960652176 | PWY0.845..superpathway.of.pyridoxal.5..phosphate.biosynthesis.and.salvage | 2.83E-06 | 9.97E-06 |
| 0.933043 | 0.905873339 | 0.960638963 | PWY.6507..4.deoxy.L.threo.hex.4.enopyranuronate.degradation | 3.62E-06 | 1.26E-05 |
| 0.932728 | 0.906240366 | 0.959805671 | PWY.7094..fatty.acid.salvage | 1.97E-06 | 7.13E-06 |
| 0.932037 | 0.906202138 | 0.958385953 | PWY.7858...5Z..dodecenoate.biosynthesis.II | 8.21E-07 | 3.32E-06 |
| 0.931269 | 0.904485575 | 0.958583893 | NAD.BIOSYNTHESIS.II..NAD.salvage.pathway.III..to.nicotinamide.riboside. | 1.53E-06 | 5.77E-06 |
| 0.931098 | 0.904586984 | 0.958158198 | DARABCATK12.PWY..D.arabinose.degradation.I | 1.13E-06 | 4.34E-06 |
| 0.9308 | 0.903689163 | 0.958394779 | PWY.7323..superpathway.of.GDP.mannose.derived.O.antigen.building.blocks.biosynthesis | 1.74E-06 | 6.35E-06 |
| 0.930695 | 0.90428483 | 0.957640846 | HEME.BIOSYNTHESIS.II.1..heme.b.biosynthesis.V..aerobic. | 8.94E-07 | 3.56E-06 |
| 0.929855 | 0.902400212 | 0.957862989 | PWY.6859..all.trans.farnesol.biosynthesis | 1.73E-06 | 6.35E-06 |
| 0.929261 | 0.903229953 | 0.955775606 | PWY0.1415..superpathway.of.heme.b.biosynthesis.from.uroporphyrinogen.III | 3.61E-07 | 1.58E-06 |
| 0.928898 | 0.901641833 | 0.956701965 | PWY.5136..fatty.acid..beta..oxidation.II..plant.peroxisome. | 1.06E-06 | 4.09E-06 |
| 0.92869 | 0.901652768 | 0.956316371 | PWY.822..fructan.biosynthesis | 8.25E-07 | 3.32E-06 |
| 0.928393 | 0.898774554 | 0.958632487 | PWY.5692..allantoin.degradation.to.glyoxylate.II | 6.16E-06 | 2.05E-05 |

| | | | | | |
|---|---|---|---|---|---|
| 0.928393 | 0.898774554 | 0.958632487 | URDEGR.PWY..superpathway.of.allantoin.degradation.in.plants | 6.16E-06 | 2.05E-05 |
| 0.928339 | 0.901777168 | 0.955430824 | PWY.7187..pyrimidine.deoxyribonucleotides.de.novo.biosynthesis.II | 4.50E-07 | 1.90E-06 |
| 0.927828 | 0.900596772 | 0.955555576 | FUC.RHAMCAT.PWY..superpathway.of.fucose.and.rhamnose.degradation | 7.05E-07 | 2.89E-06 |
| 0.927705 | 0.900948347 | 0.954906208 | COLANSYN.PWY..colanic.acid.building.blocks.biosynthesis | 4.36E-07 | 1.86E-06 |
| 0.926939 | 0.899464593 | 0.955023354 | GLYCOLYSIS.TCA.GLYOX.BYPASS..superpathway.of.glycolysis..pyruvate.dehydrogenase..TCA..and.glyoxylate.bypass | 6.88E-07 | 2.85E-06 |
| 0.926822 | 0.900235849 | 0.953983693 | PWY0.166..superpathway.of.pyrimidine.deoxyribonucleotides.de.novo.biosynthesis..E..coli. | 2.75E-07 | 1.24E-06 |
| 0.926791 | 0.899614432 | 0.954369711 | PWY.1269..CMP.3.deoxy.D.manno.octulosonate.biosynthesis | 4.74E-07 | 1.98E-06 |
| 0.925888 | 0.899697567 | 0.952564549 | PWY.6961..L.ascorbate.degradation.II..bacterial..aerobic. | 1.23E-07 | 5.96E-07 |
| 0.92587 | 0.898788062 | 0.953513855 | PWY.6531..mannitol.cycle | 3.21E-07 | 1.43E-06 |
| 0.925743 | 0.898006598 | 0.953696339 | PWY.621..sucrose.degradation.III..sucrose.invertase. | 4.21E-07 | 1.82E-06 |
| 0.925559 | 0.89715502 | 0.954593136 | KDO.NAGLIPASYN.PWY..superpathway.of..Kdo.2.lipid.A.biosynthesis | 1.01E-06 | 3.95E-06 |
| 0.925353 | 0.897862986 | 0.95329595 | PWY.7663..gondoate.biosynthesis..anaerobic. | 3.40E-07 | 1.50E-06 |
| 0.924989 | 0.897872846 | 0.952628039 | HEME.BIOSYNTHESIS.II..heme.b.biosynthesis.I..aerobic. | 2.39E-07 | 1.10E-06 |
| 0.924652 | 0.898134026 | 0.951716241 | PWY.7883..anhydromuropeptides.recycling.II | 1.15E-07 | 5.62E-07 |
| 0.924609 | 0.89805632 | 0.951674763 | PRPP.PWY..superpathway.of.histidine..purine..and.pyrimidine.biosynthesis | 1.15E-07 | 5.62E-07 |
| 0.924562 | 0.897252833 | 0.952311747 | P441.PWY..superpathway.of.N.acetylneuraminate.degradation | 2.47E-07 | 1.13E-06 |
| 0.92422 | 0.894760736 | 0.954307124 | CARNMET.PWY..L.carnitine.degradation.I | 1.59E-06 | 5.93E-06 |
| 0.923931 | 0.897518722 | 0.950854099 | PWY.5910..superpathway.of.geranylgeranyldiphosphate.biosynthesis.I..via.mevalonate. | 7.65E-08 | 3.84E-07 |
| **0.923564** | **0.896354044** | **0.951180114** | **ARGININE.SYN4.PWY..L.ornithine.biosynthesis.II** | **1.39E-07** | **6.64E-07** |

| | | | | | |
|---|---|---|---|---|---|
| 0.923453 | 0.896152859 | 0.951460195 | PWY.6608..guanosine.nucleotides.degradation.III | 1.84E-07 | 8.61E-07 |
| 0.923319 | 0.895961693 | 0.951262006 | ECASYN.PWY..enterobacterial.common.antigen.biosynthesis | 1.75E-07 | 8.26E-07 |
| 0.923156 | 0.89668361 | 0.950136188 | PWY.922..mevalonate.pathway.I..eukaryotes.and.bacteria. | 6.10E-08 | 3.19E-07 |
| 0.922653 | 0.896148361 | 0.949620102 | PWY.5837..2.carboxy.1.4.naphthoquinol.biosynthesis | 5.10E-08 | 2.80E-07 |
| 0.922338 | 0.895447049 | 0.949625336 | HEMESYN2.PWY..heme.b.biosynthesis.II..oxygen.independent. | 7.21E-08 | 3.72E-07 |
| 0.922168 | 0.895765559 | 0.949134064 | PWY.801..homocysteine.and.cysteine.interconversion | 3.99E-08 | 2.40E-07 |
| **0.92169** | **0.894984526** | **0.948858708** | **PWY.5505..L.glutamate.and.L.glutamine.biosynthesis** | **4.51E-08** | **2.63E-07** |
| 0.921515 | 0.894642098 | 0.948910099 | PWY.8178..pentose.phosphate.pathway..non.oxidative.branch..II | 5.12E-08 | 2.80E-07 |
| 0.921501 | 0.89106591 | 0.952561969 | LIPASYN.PWY..phospholipases | 1.53E-06 | 5.77E-06 |
| 0.920988 | 0.89406085 | 0.948204795 | PYRIDOXSYN.PWY..pyridoxal.5..phosphate.biosynthesis.I | 3.98E-08 | 2.40E-07 |
| 0.920553 | 0.894024572 | 0.947538474 | PWY.5861..superpathway.of.demethylmenaquinol.8.biosynthesis.I | 2.34E-08 | 1.49E-07 |
| 0.920534 | 0.893498131 | 0.948122044 | NONOXIPENT.PWY..pentose.phosphate.pathway..non.oxidative.branch..I | 4.50E-08 | 2.63E-07 |
| 0.919918 | 0.892563222 | 0.947763531 | P621.PWY..nylon.6.oligomer.degradation | 4.86E-08 | 2.75E-07 |
| 0.919484 | 0.891910226 | 0.947601238 | PPGPPMET.PWY..ppGpp.metabolism | 5.45E-08 | 2.88E-07 |
| 0.919164 | 0.892036405 | 0.946826912 | PWY66.429..fatty.acid.biosynthesis.initiation..mitochondria. | 2.91E-08 | 1.83E-07 |
| **0.9184** | **0.892061939** | **0.945285453** | **PWY.6293..superpathway.of.L.cysteine.biosynthesis..fungi.** | **8.38E-09** | **5.91E-08** |
| 0.91803 | 0.89067394 | 0.945383938 | PANTOSYN.PWY..superpathway.of.coenzyme.A.biosynthesis.I..bacteria. | 1.52E-08 | 9.83E-08 |
| 0.917973 | 0.891226758 | 0.945231081 | PWY0.301..L.ascorbate.degradation.I..bacterial..anaerobic. | 1.16E-08 | 7.75E-08 |
| 0.91719 | 0.890442058 | 0.94457051 | PANTO.PWY..phosphopantothenate.biosynthesis.I | 9.31E-09 | 6.45E-08 |

| | | | | | |
|---|---|---|---|---|---|
| **0.916649** | **0.889769073** | **0.944039675** | **P122.PWY..heterolactic.fermentation** | **8.18E-09** | **5.87E-08** |
| 0.916341 | 0.889098317 | 0.944133008 | PWY.7211..superpathway.of.pyrimidine.deoxyribonucleotides.de.novo.biosynthesis | 1.16E-08 | 7.75E-08 |
| 0.916313 | 0.889019023 | 0.944191907 | PWY0.1298..superpathway.of.pyrimidine.deoxyribonucleosides.degradation | 1.18E-08 | 7.75E-08 |
| 0.915077 | 0.88848554 | 0.942107832 | PWY.5897..superpathway.of.menaquinol.11.biosynthesis | 2.86E-09 | 2.25E-08 |
| 0.915077 | 0.88848554 | 0.942107832 | PWY.5898..superpathway.of.menaquinol.12.biosynthesis | 2.86E-09 | 2.25E-08 |
| 0.915077 | 0.88848554 | 0.942107832 | PWY.5899..superpathway.of.menaquinol.13.biosynthesis | 2.86E-09 | 2.25E-08 |
| 0.914583 | 0.887224479 | 0.942404842 | PWY.5981..CDP.diacylglycerol.biosynthesis.III | 6.47E-09 | 4.73E-08 |
| 0.913222 | 0.886900811 | 0.939969298 | PWY.5845..superpathway.of.menaquinol.9.biosynthesis | 8.99E-10 | 9.27E-09 |
| 0.91313 | 0.885933554 | 0.940550737 | FUCCAT.PWY..fucose.degradation | 2.28E-09 | 1.91E-08 |
| 0.913011 | 0.886174915 | 0.940309479 | GALACTARDEG.PWY..D.galactarate.degradation.I | 1.74E-09 | 1.60E-08 |
| 0.913011 | 0.886174915 | 0.940309479 | GLUCARGALACTSUPER.PWY..superpathway.of.D.glucarate.and.D.galactarate.degradation | 1.74E-09 | 1.60E-08 |
| 0.912434 | 0.886117735 | 0.939164567 | PWY.5862..superpathway.of.demethylmenaquinol.9.biosynthesis | 6.34E-10 | 7.08E-09 |
| 0.911759 | 0.885148396 | 0.938798001 | PWY.5838..superpathway.of.menaquinol.8.biosynthesis.I | 7.41E-10 | 8.05E-09 |
| **0.911719** | **0.884519287** | **0.939335098** | **PWY.7013...S..propane.1.2.diol.degradation** | **1.80E-09** | **1.60E-08** |
| **0.911342** | **0.884020179** | **0.939196395** | **PWY.6936..seleno.amino.acid.biosynthesis..plants.** | **1.86E-09** | **1.63E-08** |
| 0.911124 | 0.883719827 | 0.939019103 | PWY66.409..superpathway.of.purine.nucleotide.salvage | 1.91E-09 | 1.64E-08 |
| **0.91071** | **0.883678378** | **0.937975607** | **PWY.5154..L.arginine.biosynthesis.III..via.N.acetyl.L.citrulline.** | **8.90E-10** | **9.27E-09** |
| 0.910609 | 0.883261261 | 0.938463944 | GLCMANNANAUT.PWY..superpathway.of.N.acetylglucosamine..N.acetylmannosamine.and.N.acetylneuraminate.degradation | 1.29E-09 | 1.30E-08 |
| 0.910281 | 0.879721238 | 0.941397236 | PWY0.1533..methylphosphonate.degradation.I | 5.23E-08 | 2.80E-07 |

| | | | | | |
|---|---|---|---|---|---|
| 0.910281 | 0.879721238 | 0.941397236 | PWY.7807..glyphosate.degradation.III | 5.23E-08 | 2.80E-07 |
| 0.907983 | 0.879973376 | 0.935922901 | PWY.7282..4.amino.2.methyl.5.diphosphomethylpyrimidine.biosynthesis.II | 2.68E-10 | 3.53E-09 |
| 0.907689 | 0.880646805 | 0.934786659 | PWY0.1586..peptidoglycan.maturation..meso.diaminopimelate.containing. | 2.51E-10 | 3.47E-09 |
| 0.906107 | 0.879747414 | 0.932952366 | P105.PWY..TCA.cycle.IV..2.oxoglutarate.decarboxylase. | 4.53E-11 | 7.92E-10 |
| 0.905644 | 0.877999542 | 0.933555319 | PWY0.1261..anhydromuropeptides.recycling.I | 2.72E-10 | 3.53E-09 |
| 0.905358 | 0.87892049 | 0.932135927 | PWY.5840..superpathway.of.menaquinol.7.biosynthesis | 3.27E-11 | 6.57E-10 |
| **0.905058** | **0.878281633** | **0.932353223** | **P4.PWY..superpathway.of.L.lysine..L.threonine.and.L.methionine.biosynthesis.I** | **5.81E-11** | **8.98E-10** |
| 0.905058 | 0.878281633 | 0.932353223 | PWY0.781..aspartate.superpathway | 5.81E-11 | 8.98E-10 |
| 0.904974 | 0.878247275 | 0.932230716 | P164.PWY..purine.nucleobases.degradation.I..anaerobic. | 5.32E-11 | 8.92E-10 |
| 0.903779 | 0.87690119 | 0.931006475 | PWY.6305..superpathway.of.putrescine.biosynthesis | 3.74E-11 | 7.15E-10 |
| 0.903673 | 0.875409349 | 0.932439558 | PWY.7269..mitochondrial.NADPH.production..yeast. | 3.06E-10 | 3.84E-09 |
| 0.903406 | 0.87602823 | 0.930917008 | POLYISOPRENSYN.PWY..polyisoprenoid.biosynthesis..E..coli. | 4.52E-11 | 7.92E-10 |
| 0.902091 | 0.87514117 | 0.929535579 | FOLSYN.PWY..superpathway.of.tetrahydrofolate.biosynthesis.and.salvage | 2.06E-11 | 4.59E-10 |
| 0.902091 | 0.87514117 | 0.929535579 | PWY.6612..superpathway.of.tetrahydrofolate.biosynthesis | 2.06E-11 | 4.59E-10 |
| 0.901845 | 0.87483732 | 0.929251636 | PWY.7199..pyrimidine.deoxyribonucleosides.salvage | 1.57E-11 | 3.95E-10 |
| 0.901641 | 0.874726759 | 0.929007987 | PWY0.1477..ethanolamine.utilization | 1.57E-11 | 3.95E-10 |
| 0.89785 | 0.870330175 | 0.924961653 | PWY.5659..GDP.mannose.biosynthesis | 1.88E-12 | 6.31E-11 |
| 0.89681 | 0.866001727 | 0.927988464 | PROTOCATECHUATE.ORTHO.CLEAVAGE.PWY..protocatechuate.degradation.II..ortho.cleavage.pathway. | 6.28E-10 | 7.08E-09 |
| 0.895506 | 0.868633711 | 0.92279343 | GLUCARDEG.PWY..D.glucarate.degradation.I | 8.25E-13 | 3.01E-11 |

| | | | | | |
|---|---|---|---|---|---|
| 0.895474 | 0.867895239 | 0.923472712 | PWY0.1297..superpathway.of.purine.deoxyribonucleosides.degradation | 3.41E-12 | 1.06E-10 |
| 0.882976 | 0.855955079 | 0.910385067 | MET.SAM.PWY..superpathway.of.S.adenosyl.L.methionine.biosynthesis | 2.42E-15 | 1.08E-13 |
| **0.882976** | **0.855955079** | **0.910385067** | **PWY.5347..superpathway.of.L.methionine.biosynthesis..transsulfuration.** | **2.42E-15** | **1.08E-13** |
| **0.878828** | **0.851941569** | **0.906067325** | **HOMOSER.METSYN.PWY..L.methionine.biosynthesis.I** | **1.96E-16** | **1.31E-14** |
| **0.87844** | **0.851494639** | **0.905740391** | **METSYN.PWY..superpathway.of.L.homoserine.and.L.methionine.biosynthesis** | **1.86E-16** | **1.31E-14** |
| 0.878269 | 0.850799394 | 0.905992406 | PHOSLIPSYN.PWY..superpathway.of.phospholipid.biosynthesis.I..bacteria. | 4.62E-16 | 2.65E-14 |
| **0.876771** | **0.849668076** | **0.904151444** | **P461.PWY..hexitol.fermentation.to.lactate..formate..ethanol.and.acetate** | **8.14E-17** | **1.31E-14** |
| 0.876661 | 0.849235545 | 0.904377599 | PWY4FS.7..phosphatidylglycerol.biosynthesis.I..plastidic. | 1.90E-16 | 1.31E-14 |
| 0.876661 | 0.849235545 | 0.904377599 | PWY4FS.8..phosphatidylglycerol.biosynthesis.II..non.plastidic. | 1.90E-16 | 1.31E-14 |
| 0.873022 | 0.846220062 | 0.900056733 | RHAMCAT.PWY..L.rhamnose.degradation.I | 4.49E-18 | 1.81E-15 |

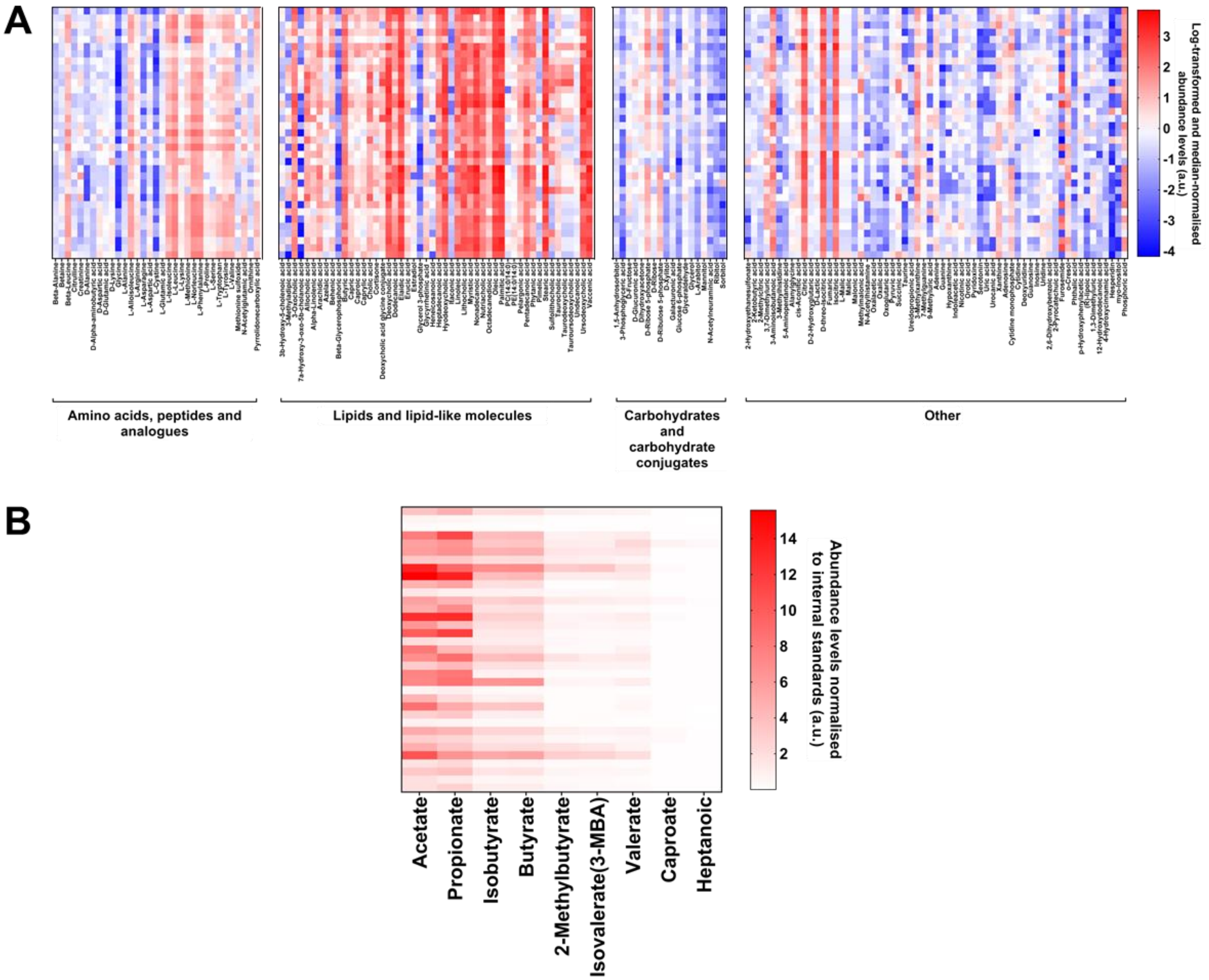
A
Log-transformed and median-normalised abundance levels (a.u.)
3
2
1
0
-1
-2
-3
-4
Amino acids, peptides and analogues
Lipids and lipid-like molecules
Carbohydrates and carbohydrate conjugates
Other
B
Abundance levels normalised to internal standards (a.u.)
14
12
10
8
6
4
2
Acetate
Propionate
Isobutyrate
Butyrate
2-Methylbutyrate
Isovalerate(3-MBA)
Valerate
Caproate
Heptanoic

## eFigure 7. Metabolite profiles of cognitively impaired residents of long-term aged care facilities.

Heatmaps illustrating the detected levels of **A**) 165 polar metabolites including amino acids, peptides and analogues, lipids and lipid-like molecules, carbohydrates and carbohydrate conjugates, and other metabolites (organic acids, organoheterocyclic compounds, nucleosides, nucleotides and analogues, benzenoids, medium-chain fatty acids, phenylpropanoids and polyketides, organic oxygen compounds, and inorganic compounds), and **B**) nine short-chain fatty acids.

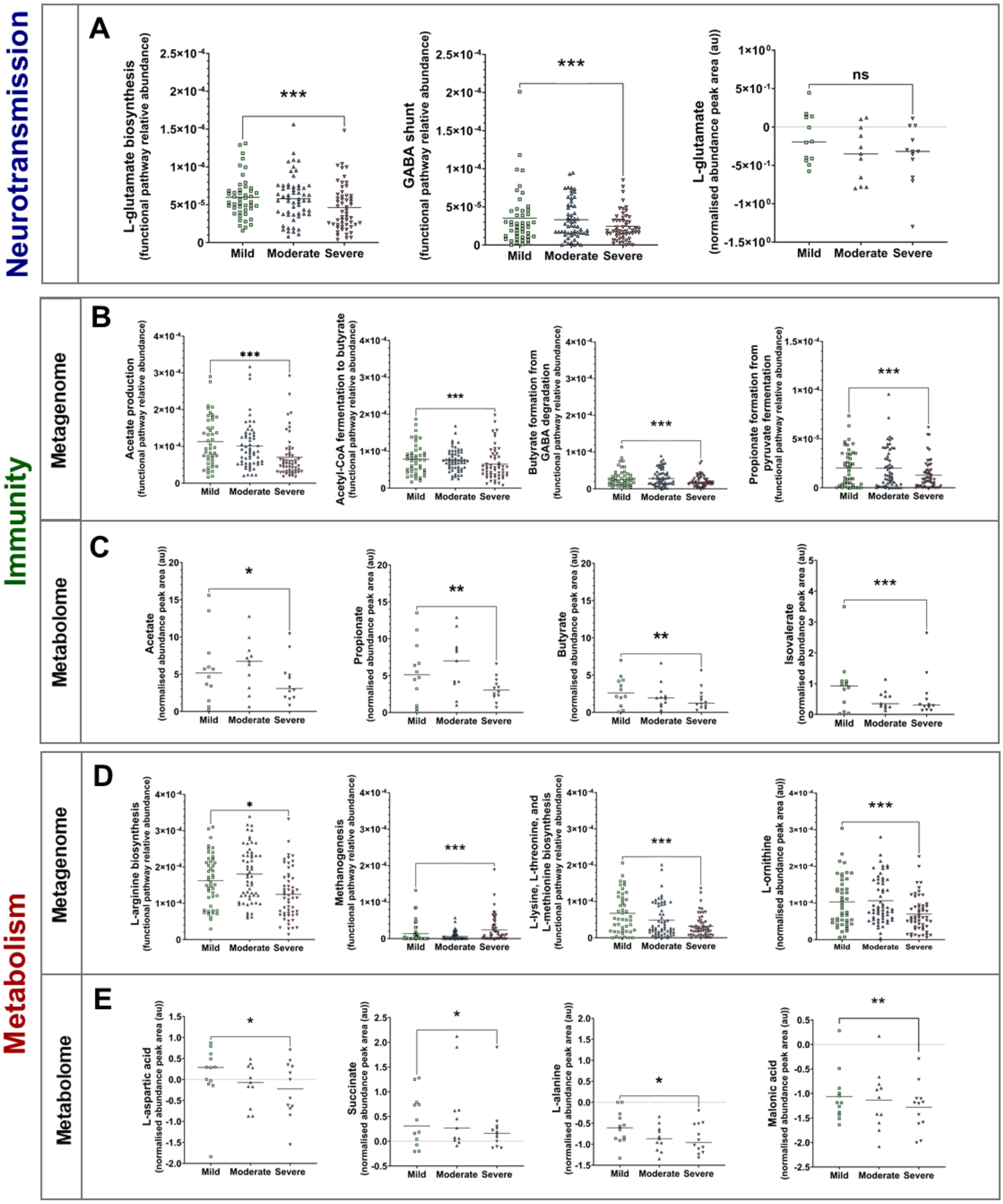


**eFigure 8. Specific functional differences relating to neurotransmission, immunity, and metabolism in the gut microbiome of residents of long-term aged care by cognitive impairment.**

Metagenomic and metabolomic profiling of microbiome functional capacity and output for long-term aged care residents with cognitive impairment in relation to neuronal communication (**A**), immunity (**B-C**), and metabolism (**D-E**). Categorical plots illustrate significance of multivariate analysis between mild and severe cognitive impairment, where mild was the reference group. ns=not significant; *q<0.05; **q<0.01; ***q<0.001 for adjusted p-values following FDR correction. Pathways: mild, n=46; moderate, n=58; severe, n=55. Metabolites: mild, n=12; moderate, n=11; severe, n=12.

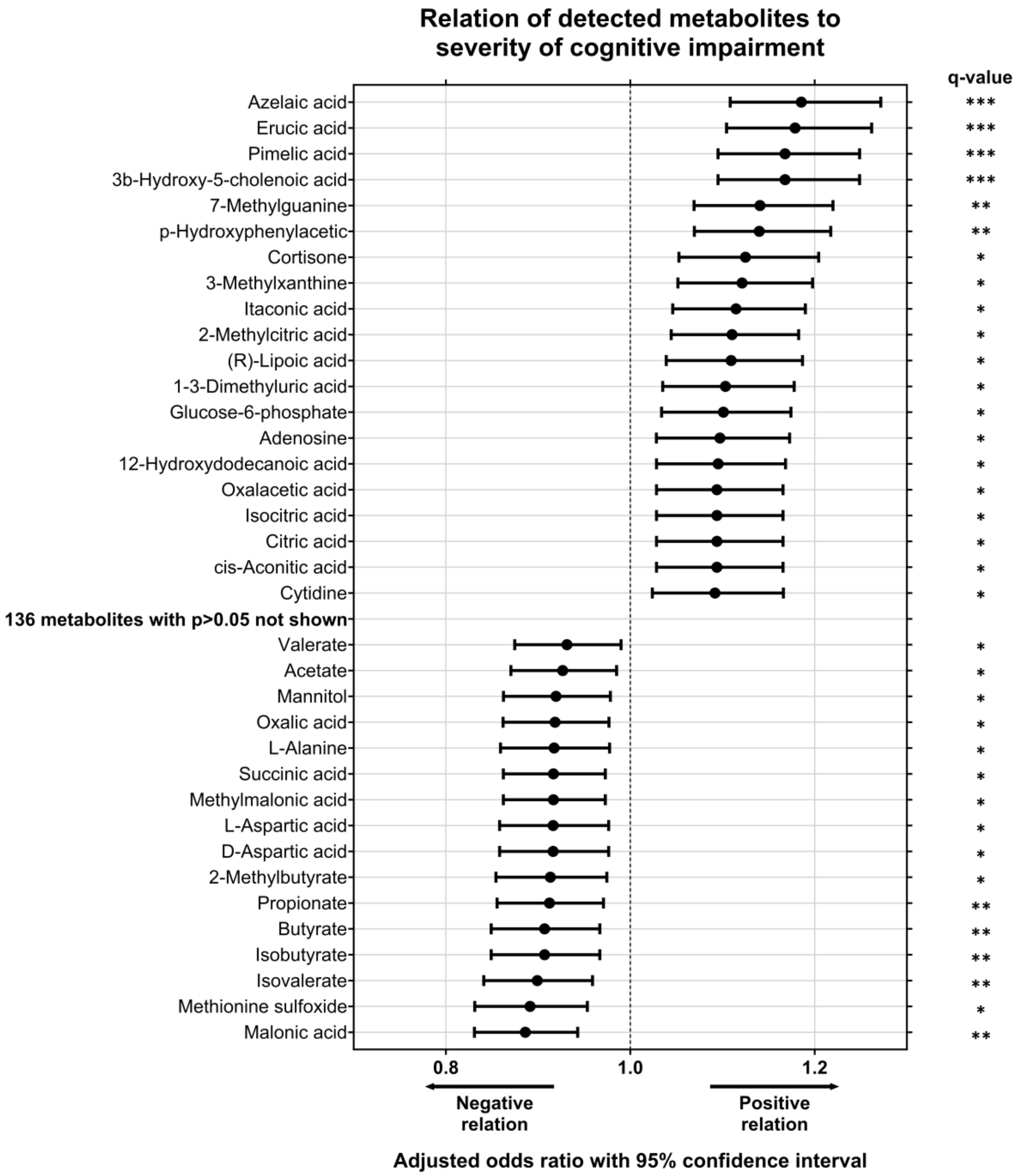


## eFigure 9. Metabolite differences in the gut microbiome of residents of long-term aged care by cognitive impairment.

All metabolites detected with significant differences by CI severity include short-chain fatty acids and polar metabolites. Odds ratio and 95% confidence interval of effect of cognitive impairment severity on detected metabolite abundance levels. Performed by multivariate analysis, adjusting for time since cognitive impairment assessment, age, sex, antibiotic use, proton pump inhibitor use, opioid use, laxative use, recorded medical history, meal texture, and liquid texture. n=12 mild; n=11 moderate; n=12 severe. *q<0.05; **q<0.01; ***q<0.001 for adjusted p-values following FDR correction.

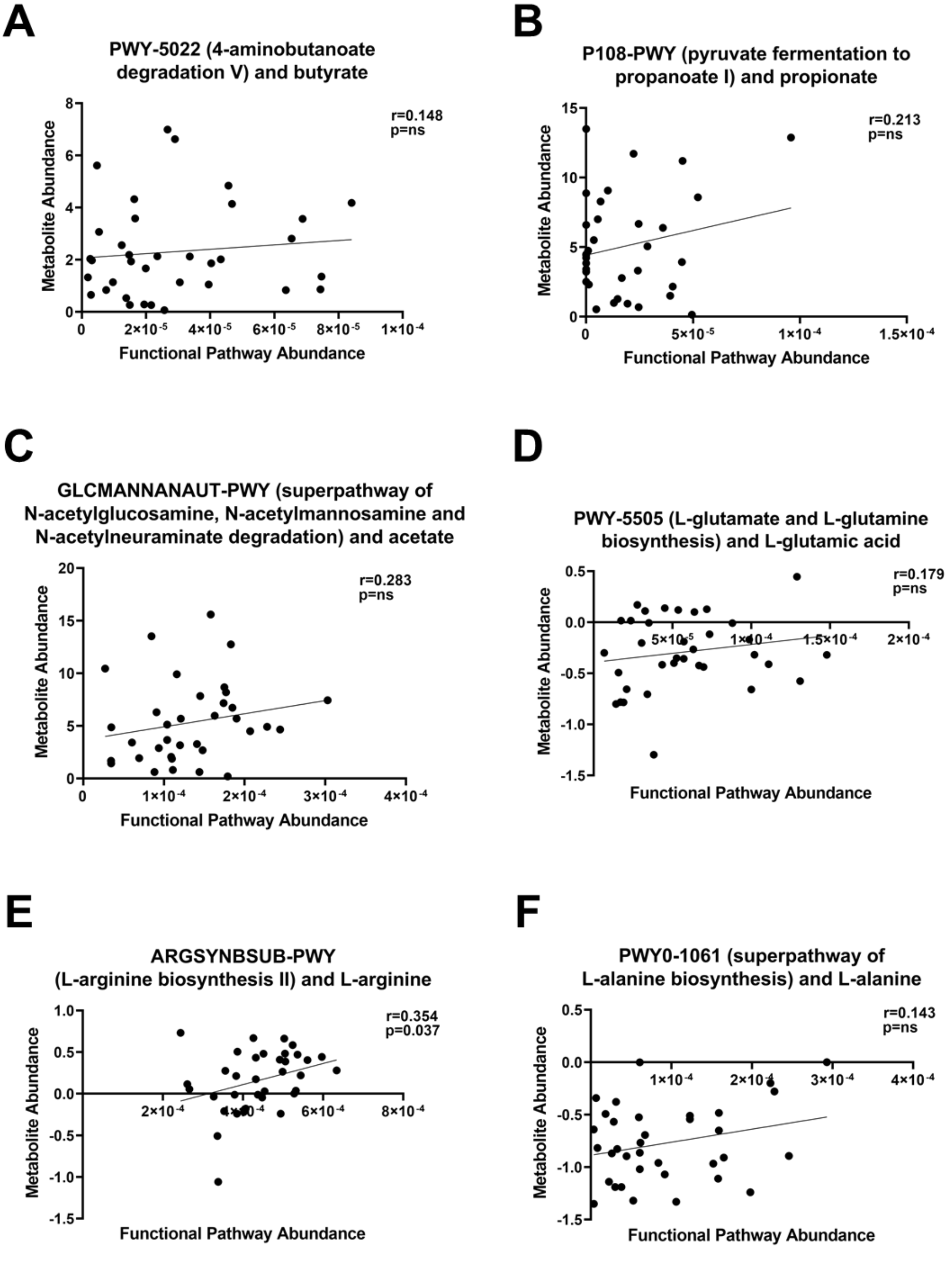


**eFigure 10. Microbial functional capacity correlates with detected metabolite levels in the gut microbiome of residents in long-term aged care with cognitive impairment.**

Correlations of the relative abundance of microbial functional capacity for mapped metagenomic pathways at the whole community level with direct metabolites detected in stool samples of the CI cohort, determined with Spearman's correlation. n=12 mild; n=11 moderate; n=12 severe.
